# Preparation and Characterization of Alginate‐Based Bioinks for Three‐Dimensional Bioprinting of Cell‐Laden Constructs

**DOI:** 10.1002/cpz1.70290

**Published:** 2025-12-28

**Authors:** Nuraina Anisa Dahlan, Farinaz Ketabat, Kathryn Avery, Xavier L. Tabil, Samira Khoz, Elise Altarriba, Neeraj Dhar, Xiongbiao Chen

**Affiliations:** ^1^ Vaccine and Infectious Disease Organization University of Saskatchewan Saskatoon Canada; ^2^ Division of Biomedical Engineering, College of Engineering University of Saskatchewan Saskatoon Canada; ^3^ Department of Mechanical Engineering, College of Engineering University of Saskatchewan Saskatoon Canada; ^4^ Department of General Engineering, Institut Catholique d'Arts et Métiers de Lille Lille Catholic University Lille France; ^5^ Respiratory Research Centre, College of Medicine University of Saskatchewan Saskatoon Canada; ^6^ Department of Biochemistry, Microbiology and Immunology University of Saskatchewan Saskatoon Canada; ^7^ Vaccinology and Immunotherapeutics, School of Public Health University of Saskatchewan Saskatoon Canada

**Keywords:** 3D bioprinting, alginate, bioink, extrusion, tissue engineering

## Abstract

Biomaterial‐based bioinks are increasingly utilized in bioprinting to engineer three‐dimensional (3D) constructs with living cells for tissue engineering and disease modeling. Among various bioinks explored, alginate‐based formulations stand out due to their good biocompatibility, mild gelation conditions, tunable mechanical properties, and ease of crosslinking via divalent cations such as calcium. Despite their widespread use, standardized protocols for preparing alginate‐based bioinks and characterizing bioprinted constructs have not been well documented. Our laboratory has developed and validated reproducible methods for preparing a variety of alginate‐based bioinks and printing cell‐laden constructs tailored for diverse applications. In this article, we present detailed step‐by‐step protocols covering bioink preparation and rheological characterization, extrusion‐based bioprinting of cell‐laden constructs, post‐printing culture and co‐culture techniques, printability assessment, and live/dead and immunofluorescence assays. These protocols serve as a standardized framework for the fabrication and characterization of 3D bioprinted alginate‐based cell‐laden constructs, thereby facilitating translational research in tissue engineering, disease modeling, and preclinical therapeutic development. © 2025 The Author(s). Current Protocols published by Wiley Periodicals LLC.

**Basic Protocol 1**: Bioink preparation

**Basic Protocol 2**: Bioink characterization using rheology

**Basic Protocol 3**: Scaffold design and bioprinting

**Support Protocol**: 3D‐printing parameter determination

**Basic Protocol 4**: Printability and cell viability analyses, and immunofluorescence assay

## INTRODUCTION

In three‐dimensional (3D) bioprinting, biomaterials‐based bioinks are widely used to fabricate 3D tissue constructs (or tissue models) embedded with living cells for tissue engineering and disease modeling. Bioink goes beyond simply creating shapes, it must provide cell‐friendly environments and structural support for encapsulated cells to grow, interact, and remodel (Chen, 2025a). 3D bioprinting is a technique that leverages a computer‐aided design (CAD) model to fabricate in vivo‐like hierarchical cell structure (Chen, 2025b). The development of effective 3D microtissue models represents a significant advancement beyond the gold standard of two‐dimensional (2D) cell culture. While 2D culture systems have long served as a foundational tool in biomedical research (Ning et al., 2016), these systems fall short in replicating the complex cellular architecture, mechanical cues, and biochemical gradients often found in vivo. In contrast, 3D microtissue models offer enhanced physiological relevance with potential to reduce dependence on animal models.

Alginate‐based bioinks are distinguished by their rapid ionic gelation, tunable mechanical properties, shear thinning properties for printability, and ability to mimic the native extracellular matrix (ECM). It is a linear anionic polysaccharide extracted from brown seaweeds that can be smoothly extruded through bioprinter nozzles. Alginate undergoes rapid gelation in the presence of divalent cations such as calcium (Ca^2+^) to effectively preserve the printed geometry (Lee & Mooney, 2012). Despite its widespread use in engineering 3D tissue scaffolds, alginate has limited long‐term structural stability due to its naturally derived origin and inherently lacks cell adhesion binding sites and mechanical properties to maintain shape fidelity and promote tissue maturation. Often, alginate is blended with other active biomolecules to improve its printability, mechanical performance, and biological properties. There are a variety of other biocompatible materials, e.g., collagen, gelatin, and decellularized ECM, to enhance alginate biocompatibility and provide tissue‐specific cues that eventually improve cell infiltration and regeneration (Blanco‐Fernandez et al., 2022; Catoira et al., 2019; Jain et al., 2021; Wang et al., 2024; Yeleswarapu et al., 2023). Although alginate‐based bioprinting has been widely reported, detailed protocols for preparing alginate‐based bioinks and characterizing bioprinted constructs remain insufficiently documented. In our lab, we have been working extensively on developing reliable protocols for printing alginate‐based bioinks, addressing challenges including achieving precise print resolution and enhancing cell viability and recovery post‐printing (Dahlan et al., 2025; Ketabat et al., 2023; Mohabatpour et al., 2022; Sadeghianmaryan et al., 2022; Sarker et al., 2019; Zimmerling, Aubrey, et al., 2024; Zimmerling, Boire, et al., 2024; Zimmerling, Sunil, et al., 2024; Zimmerling, Zhou, et al., 2024).

**Figure 1:**
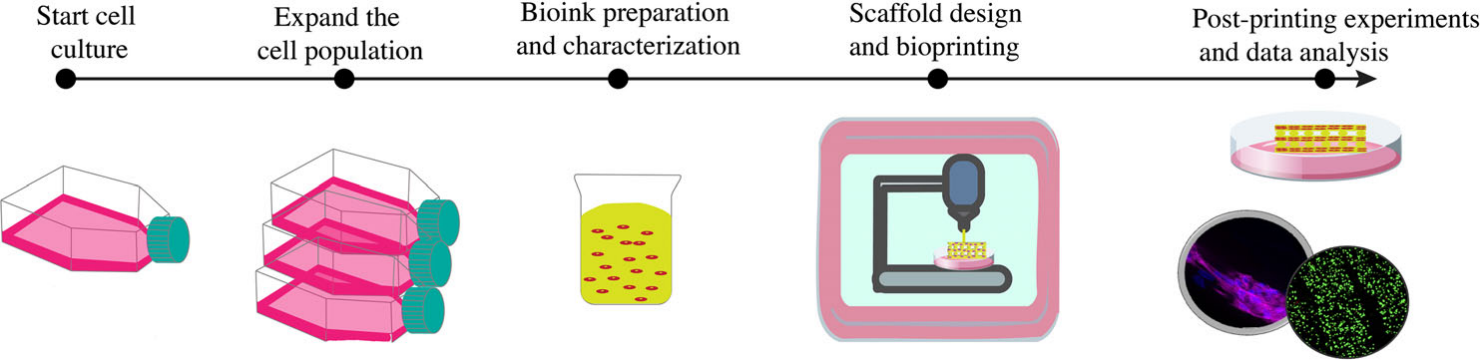
Overview of the protocols. Basic Protocol 1 covers the cell culture/expansion and bioink preparation; Basic Protocol 2 outlines the characterization of bioink using rheology; Basic Protocol 3 covers the design and bioprinting of scaffolds/constructs; Basic Protocol 4 describes the post‐printing characterization by printability, cell viability, and immunostaining assay.

The protocols in this article present a comprehensive workflow encompassing cell culture and expansion, alginate bioink preparation, scaffold or construct design and bioprinting, as well as post‐printing characterizations and data analysis, as illustrated in Figure 1. Basic Protocols 1 and 2 describe the culturing of representative cells, specifically human umbilical vein endothelial cells (HUVECs), along with the preparation of alginate‐based bioinks, and rheological characterization of bioinks. Basic Protocol 3 details the preparation for 3D printing and bioprinting process, including the selection or determination of printing parameters. To complement these steps, Support Protocol provides the preliminary printing parameter determination and fine‐tuning printing parameters. We recommend conducting preliminary tests with cell‐free hydrogel inks to screen candidates and optimizing the printing parameters or conditions before transitioning to cell‐laden bioinks. This approach facilitates troubleshooting and helps reduce material and cell‐related costs. Basic Protocol 4 present methods to assess printability, seed cells on printed constructs, conduct live/dead staining, and perform imaging and quantitative analysis using ImageJ software. We also include a modified immunostaining protocol tailored for cell‐laden hydrogel constructs.

## Basic Protocol 1 BIOINK PREPARATION

This protocol provides step‐by‐step instructions for preparing an alginate‐based bioink. It also includes complete instructions for culturing HUVECs used as an adherent cell model. The protocol is organized into two sections:

Steps 1 to 45 describe the procedures for seeding, maintaining and passaging HUVECs to obtain desired cell densities for bioink preparation.
Steps 46 to 75 detail the bioink preparation process for high cell densities to achieve a homogeneous cell‐laden alginate‐bioink for bioprinting.

*NOTE*: Always label the culture flask with the cell name, passage number, date, and your initials.

*NOTE*: Sterilize all materials, e.g., culture flasks, serological pipettes, pipette pump, with 70% ethanol before placing them in the biological safety cabinet (BSC).

*NOTE*: Cell‐related procedures must be conducted inside a BSC to prevent contamination and maintain a sterile working environment.

*CAUTION*: It is not recommended to warm the medium in a water bath, as this can cause some components to degrade. Alternatively, allow the medium come to room temperature naturally before use.

### Materials

HUVEC complete cell culture medium (see recipe)
HUV‐EC‐C (HUVEC) (ATCC, cat. no. CRL‐1730)
70% ethanol
Gibco TrypLE Express enzyme (1×), phenol red (Fisher Scientific, cat. no. 12605010); store at 4°C
Gibco phosphate‐buffered saline (PBS), pH 7.4 (Fisher Scientific, cat. no. 10010023); store at room temperature
Gibco trypan blue solution, 0.4% (Fisher Scientific, cat. no. 15250061); store at room temperature
Alginic acid sodium salt from brown algae, medium viscosity (Sigma‐Aldrich, cat. no. A2033); store at 4°C
Porcine gelatin, type A, gel strength ∼300 g bloom (Sigma‐Aldrich, cat. no. G1890); store at room temperature
0.9% NaCl solution (see Current Protocols, 2006)

Biological safety cabinet (BSC)
T75 culture flask (Corning, cat. no. 430641U)
Serological pipettes
Pipette controller
15‐ml conical centrifuge tubes (Falcon, cat. no. 14‐959‐49B)
Water bath
Centrifuge
Cell culture microscope
Cell culture incubator, 37°C, 5% CO_2_
Hemocytometer or automated cell counter
Autoclave
Beakers
Spatula, micro, tapered, 8‐in. (Fisher Scientific, cat. no. 21‐401‐10)
Magnetic stir bars
Weighing papers
50‐ml conical centrifuge tubes (Falcon, cat. no. 14‐532‐22)
Scale
Parafilm
Magnetic stirring hot plate
20‐, 200‐, and 1000‐µl micropipettes and tips
Sterile printing syringe for bioprinting or syringe barrels (Sigma‐Aldrich, cat. no. 928879)

#### Culturing HUVECs for cell expansion

##### Seeding cells

**Figure 2:**
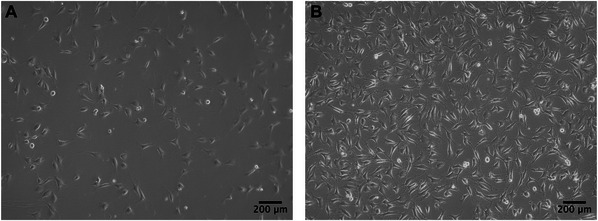
HUVECs at (**A**) low and (**B**) high densities maintained in a T75 flask.

1 Prepare the HUVEC complete culture medium and let it warm to room temperature.
2 Place T75 culture flask, 10‐ml serological pipettes, and a pipette controller in the BSC. See Notes above.
3 Add 9 ml of pre‐warmed complete HUVECs culture medium to a 15‐ml Falcon tube.
4 Remove a frozen cryovial of HUVECs from liquid nitrogen storage and thaw the vial gently and rapidly using a 37°C water bath without submerging the cap. Leave a small chunk of frozen cell crystal.
5 Wipe the vial with 70% ethanol.
6 Gently mix the contents to dissolve the remaining ice crystal and transfer the contents to the Falcon tube in step 3.
7 Add 9 ml of complete culture medium to the Falcon tube and centrifuge 5 min at 300 × *g*, room temperature.
8 Discard the supernatant inside the BSC.
9 Disperse the pellet in 1 ml culture medium and transfer it to a T75 flask containing 10 to 15 ml of fresh medium, keeping the flask in an upright (vertical) position inside the BSC.
10 Rock the flask (gently) side to side and front to back to evenly distribute the cells across the surface.
11 Check the cells under the microscope to ensure even distribution.
12 Transfer the flask to the cell culture incubator at 37°C and 5% CO_2_.
13 Do not disturb the flask for at least 16 hr.
14 Change the medium after 24 hr and check the confluency every 48 hr, as shown in Figure 2A‐B.

15 When the culture reaches ∼90% confluency, proceed to either passage cells or freeze them down.

###### Changing medium

16 Warm the HUVEC complete culture medium to room temperature. See Caution above.
17 Retrieve T75 flask from the incubator.
18 Check the cell morphology and confluency under the microscope.
19 Place the flask in the BSC. See Notes above.
20 Remove the medium using a 10‐ml serological pipette by tilting the flask to one corner and collecting the spent medium. Avoid contact with the flask walls or bottom. Do not touch the pipette against the mouth of the flask.
21 Add 10 ml of fresh, pre‐warmed HUVEC complete medium to the corner of the flask.
22 Distribute the medium evenly. This step is similar to step 10.
23 Check the condition of the cells under the microscope.
24 Place the flask in the cell culture incubator.

###### Passaging cells

25 Warm HUVEC complete culture medium, TrypLE Express, and 1× PBS to room temperature.
26 Retrieve the T75 flask containing HUVECs from the cell culture incubator.
27 Check the cell morphology and confluency under the microscope to ensure the cells are ∼90% confluent.
28 Transfer the flask into the BSC.
29 Aspirate the medium from the corner of the flask using a 10‐ml serological pipette.
30 Add 5 ml of 1× PBS to the bottom corner of the flask and gently swirl the liquid across the surface and remove it promptly.
31 Add 2 ml of TrypLE Express to the medium and distribute it evenly. Leave the liquid to cover the surface for 1 min in the BSC and transfer the flask to the incubator for 6 to 7 min.
32 Retrieve the flask from the incubator and check cell rounding or detachment under the microscope.
33 Place the flask in the BSC.
34 Add 2 ml fresh medium (equal volume) and neutralize the trypsinized cells.
35 Gently disperse the medium by pipetting over the cell layer surface several times to ensure >95% cell recovery. Transfer the cell suspension into a 15‐ml Falcon tube.
  Gentle pipetting helps to dislodge and recover a high percentage of cells. This is also useful to break any large clumps of cells that might form after dissociation or centrifugation.
36 If needed, examine the flask under the microscope to ensure <5% of cells remain. Add 2 to 4 ml fresh medium to collect any remaining cells and add to the same Falcon tube in step 34.
37 Centrifuge 5 min at 300 × *g*, room temperature.
38 After centrifugation, carefully aspirate the supernatant without disturbing the cell pellet.
39 Resuspend the cell pellet in 1 ml of fresh complete cell culture medium.
40 Count cells by mixing 10 µl cell suspension with 10 µl trypan blue solution. Load the stained cells into a hemocytometer or automated cell counter.
41 Based on the total cell number, transfer cell suspension at a density of 2500 viable cells/cm^2^ into a new T75 flask.
  Alternatively, cells can be routinely passaged at a 1:3 ratio. In our lab, cells are typically maintained for up to 1‐month‐post‐thaw. A new vial should be thawed either after this 1‐month period or earlier if the cells show noticeably slower growth or morphological changes.
42 Add fresh complete cell culture medium to reach a final volume of 10 ml and gently disperse the cell suspension to cover all surfaces.
43 Maintain cells in the cell culture incubator and confirm that they are tested negative for mycoplasma using the agar‐and‐broth or polymerase chain reaction (PCR) methods (Siegl et al., 2023; Young et al., 2010).
44 Repeat steps 13 and 15.
45 Change medium every 2 to 3 days. Refer to “Changing medium” above.

#### Alginate‐based bioink preparation

##### Day 1: Hydrogel mix preparation

Fresh hydrogel mix must be prepared 1 day prior to bioprinting or rheological analyses to ensure appropriate cell viability, printability, and consistency in material properties. It is recommended to prepare the hydrogel mix at ∼70% higher concentration to account for subsequent dilution with the cell suspension in culture medium. For example, if the final desired concentration is 3% (w/v) alginate and 1% (w/v) gelatin, prepare the initial hydrogel at 4.28% (w/v) alginate and 1.43% (w/v) gelatin. Then, mix the hydrogel with the cell suspension (prepared in culture medium) in a 70:30 ratio to achieve the target final concentrations.

46 Prepare a 0.9% NaCl solution in deionized water (saline solution) and sterilize it by autoclaving or using a commercially available sterile saline solution.
47 Autoclave all equipment, including beakers, spatulas, magnetic stir bars, and weighing papers before use.
48 Disinfect all containers holding polymers (e.g., alginate, gelatin) by spraying them with 70% ethanol and placing them inside the BSC.
  Ensure that all containers containing polymer powders (e.g., alginate, gelatin) are only opened inside a BSC. Opening them outside the BSC compromises sterility.
  Although the polymers used were not purchased as sterile, no contamination issues have been observed in our lab when following this sterilization and handling protocol.
49 Transfer a small amount of polymer powder into a 50‐ml sterile Falcon tube. Take the tube to a scale located in a closed chamber that has been thoroughly disinfected with 70% ethanol. Weigh the required amount using autoclaved weighing paper and wrap the weighed portion in another sheet of autoclaved weighing paper for transfer.
50 Bring the weighed polymer powder into the BSC.
51 In the BSC, fill a sterile beaker with the required volume of autoclaved 0.9% saline solution.
52 Disperse the weighed gelatin powder into the saline‐filled beaker.
53 Place a sterile magnetic stir bar into the beaker.
54 Cover the beaker securely with two layers of Parafilm to prevent contamination and potential evaporation.
55 Place the beaker on a magnetic stirring hot plate set to 50° to 60°C. Stir continuously until the gelatin is fully dissolved (1 to 2 hr).
56 If preparing alginate‐based hydrogels (e.g., 3% (w/v) alginate and 1% (w/v) gelatin), always dissolve the less concentrated polymer first. In this case, gelatin should be dissolved before adding alginate.
57 Weigh the required amount of alginate powder following the same procedure used for gelatin (i.e., using autoclaved weighing paper in a disinfected balance chamber).
58 Once the gelatin is fully dissolved, transfer the beaker back into the BSC.
59 Gradually disperse the weighed alginate powder into the gelatin solution in the beaker.
60 Using an autoclaved spatula, thoroughly mix the alginate into the solution until it is completely dispersed, and no powder residue remains on the spatula or in the beaker. This typically takes 15 to 30 min.
61 Re‐seal the beaker with two layers of Parafilm.
62 Place the beaker back onto the stirring hot plate. Set the stirrer to the lowest speed sufficient to gently rotate the magnetic stir bar.
63 Allow the hydrogel mixture to stir for at least 2 hr.
  Do not leave the hydrogel on the heated stirrer overnight.
64 After stirring, bring the hydrogel beaker back into the BSC.
65 Using an autoclaved spatula, transfer the hydrogel mixture into a sterile 50‐ml Falcon tube.
66 Centrifuge the tube 5 to 10 min at 300 × *g*, room temperature, until the hydrogel appears clearer and a visible layer of bubbles forms on the top.
67 Return the hydrogel to the BSC. Carefully remove the bubbly layer using an autoclaved spatula.
68 Store the hydrogel at 4°C overnight.
  Overnight refrigeration is necessary to allow the hydrogel to recover from shear stress introduced during mixing and centrifugation, ensuring greater printability during bioprinting.

###### Day 2: Preparing cell‐laden bioink

69 Dissociate the cells using the method described in steps 25 to 36.
  Since the alginate/gelatin hydrogel was prepared at higher concentrations (to account for dilution with cell suspension), proceed with the following calculations to determine the appropriate mixing ratio to reach your target final concentrations (e.g., 3% alginate and 1% gelatin)
  Example calculation (Eq. 1): If you count 24 million cells and require a final concentration of 6 million cells/ml, the total volume of your bioink should be:
(1) $$\begin{array}{ccc} & & \;\;\;\;\;\;\;\;\frac{\mathrm{Total}\;\mathrm{number}\;\mathrm{of}\;\mathrm{cells}\;\left(24\;\times 10^{6}\right)}{\mathrm{Numb}\mathrm{er}\;\mathrm{of}\;\mathrm{cells}\;\mathrm{per}\;\mathrm{ml}\;\left(6\;\times 10^{6}\right)}=4\;\mathrm{ml}\;\left(\mathrm{total}\;\mathrm{volu}\mathrm{me}\;\mathrm{of}\;\mathrm{bioi}\mathrm{nk}\right)\\ & & \;\;\;\;\;\;\;\;\;4.28\%\;\left(\mathrm{Alg}\;4.28\%\right)\times \mathrm{volume}=3\%\;\left(\mathrm{Alg}3\%\right)\times \;4\;\mathrm{ml}\\ & & \;\;\;\;\;\;\;\;\;\mathrm{required}\;\mathrm{volume}\;\mathrm{of}\;\mathrm{Alg}\;4.28\%=2.80\;\mathrm{ml}\\ & & \;\;\;\;\;\;\;\;\;4\;\mathrm{ml}\;\mathrm{total}\;\mathrm{volume}\;\mathrm{of}\;\mathrm{bioink}-2.80\;\mathrm{ml}\;\mathrm{of}\;\mathrm{Alg}\;4.28\%\\ & & \;\;\;\;\;\;\;\;\;=1.2\;\mathrm{ml}\;\left(\mathrm{the}\;\mathrm{required}\;\mathrm{volume}\;\mathrm{of}\;\mathrm{culture}\;\mathrm{medium}\;\mathrm{in}\;\mathrm{which}\;\mathrm{to}\;\mathrm{disperse}\;\mathrm{the}\;\mathrm{cells}\right) \end{array}$$
70 After counting (see step 40), centrifuge the cell suspension 5 min at 300 × *g*, room temperature.
71 Carefully discard the supernatant without disturbing the cell pellet.
72 Disperse the cell pellet in the pre‐calculated volume of complete culture medium (e.g., 1.2 ml in this example).
  It is also helpful to use culture medium containing phenol when preparing the cell suspension. The pink color helps to visually confirm that the cells are evenly blended into the hydrogel.
73 Transfer the calculated volume of hydrogel prepared based on step 69 (e.g., 2.80 ml) into a sterile printing syringe for bioprinting, or into a Falcon tube for rheological analyses, using a wide‐bore 1‐ml pipette tip.
  If a wide‐bore tip is unavailable, use a regular pipette tip with the end cut off using autoclaved scissors to improve flow, as these hydrogels could be highly viscous.
  A manual mixing method may also be suitable for viscous materials and low cell densities (<1 × 10^6^ cells/ml) as this technique minimizes bubbles formation. Blend the mixture slowly and gently using a flat spatula in one direction. Avoid creating bubbles.
74 Set the pipette controller to ≤500 µl when handling the hydrogel to prevent it from being drawn into the controller due to its viscosity.
  After a few transfers, the resistance may increase; take care to avoid backflow into the pipette controller.
75 Using the same modified pipette tip, transfer the resuspended cell suspension into the syringe containing the hydrogel. Mix very gently by pipetting up and down multiple times until a homogeneous cell‐hydrogel mixture is achieved.
  Alternatively, transfer of bioink into the syringe can be done using a gravity method by allowing the hydrogel to flow passively into the syringe. Any leftover material can be gently scrapped out using a flat spatula.

## Basic Protocol 2 BIOINK CHARACTERIZATION USING RHEOLOGY

This protocol provides a detailed guide for characterizing bioink candidates using rheological measurements. It outlines the procedures for conducting rheological tests to evaluate hydrogel properties, including shear‐thinning behavior and viscosity. The protocol is divided into two sections:

Steps 1 to 13 cover the instrument set up, cone selection and calibration.
Steps 14 to 37 outline sample loading, experiment set up, test parameters, and data acquisition.

*CAUTION: If the pressure is not set correctly, the bearing and instrument could be damaged. To prevent this, the regulator is attached to an alarm that visibly and audibly alerts the operator if the pressure is too low. Testing should be discontinued, or the backup pressure line should be opened if in the middle of a test*.

### Materials

dH_2_O
70% ethanol
Hydrogel ink and bioink from Basic Protocol 1

Discovery hybrid rheometer (TA Instruments, Series HR 20)
TRIOS v5.7.2.101 software (software supplied by TA Instruments)
Disposable paper towels
Polystyrene antistatic weighing boats (Fisher Scientific, cat. no. 08‐732‐112)

#### Rheology

This protocol is based on our established work by Zimmerling, Sunil, et al. (2024) and Zimmerling, Zhou, et al. (2024).

##### Setting up the Discovery‐series HR 20

**Figure 3:**
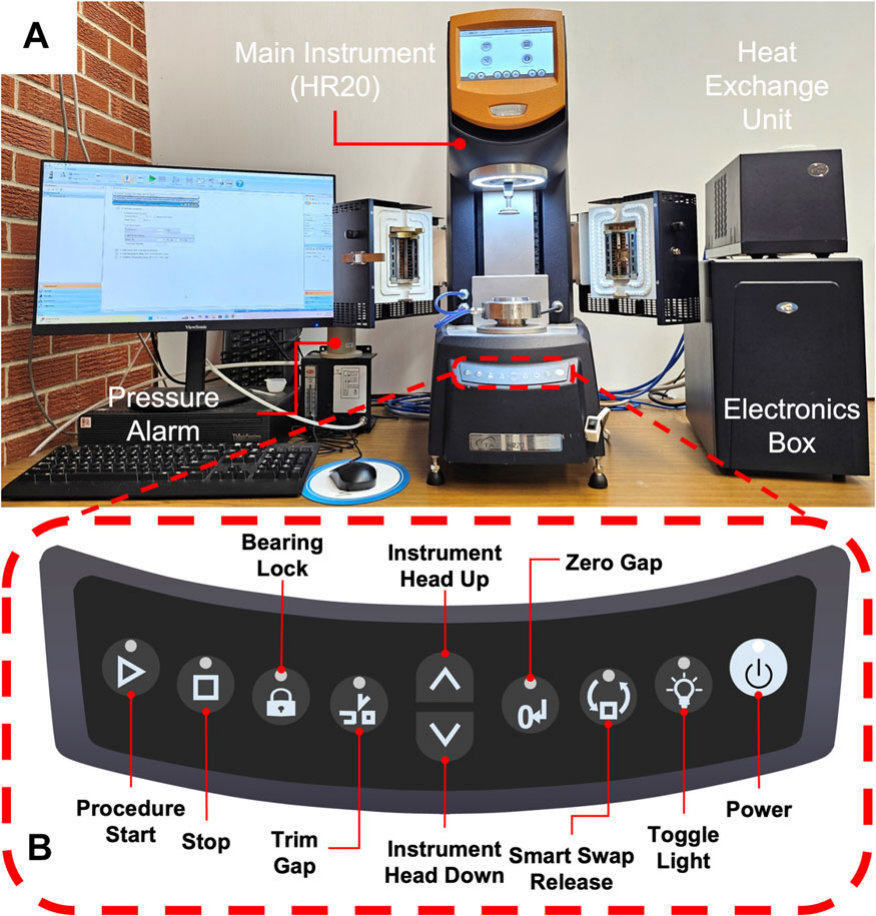
(**A**) Current HR 20 setup with the main components labeled. Not visible is the pressure regulator, which is behind the computer screen. (**B**) Front panel buttons of the main instrument. The most used buttons will be the Power, Toggle Light, Instrument Head Up/Down, and Bearing Lock buttons.

1 Check that a pneumatic pressure of 2 bar (∼30 psi) is being supplied to the rheometer and adjust the regulator if necessary. Turn on the switch attached to the rheometer that is connected to a pressure sensor (Fig. 3A). See Caution above.

**Figure 4:**
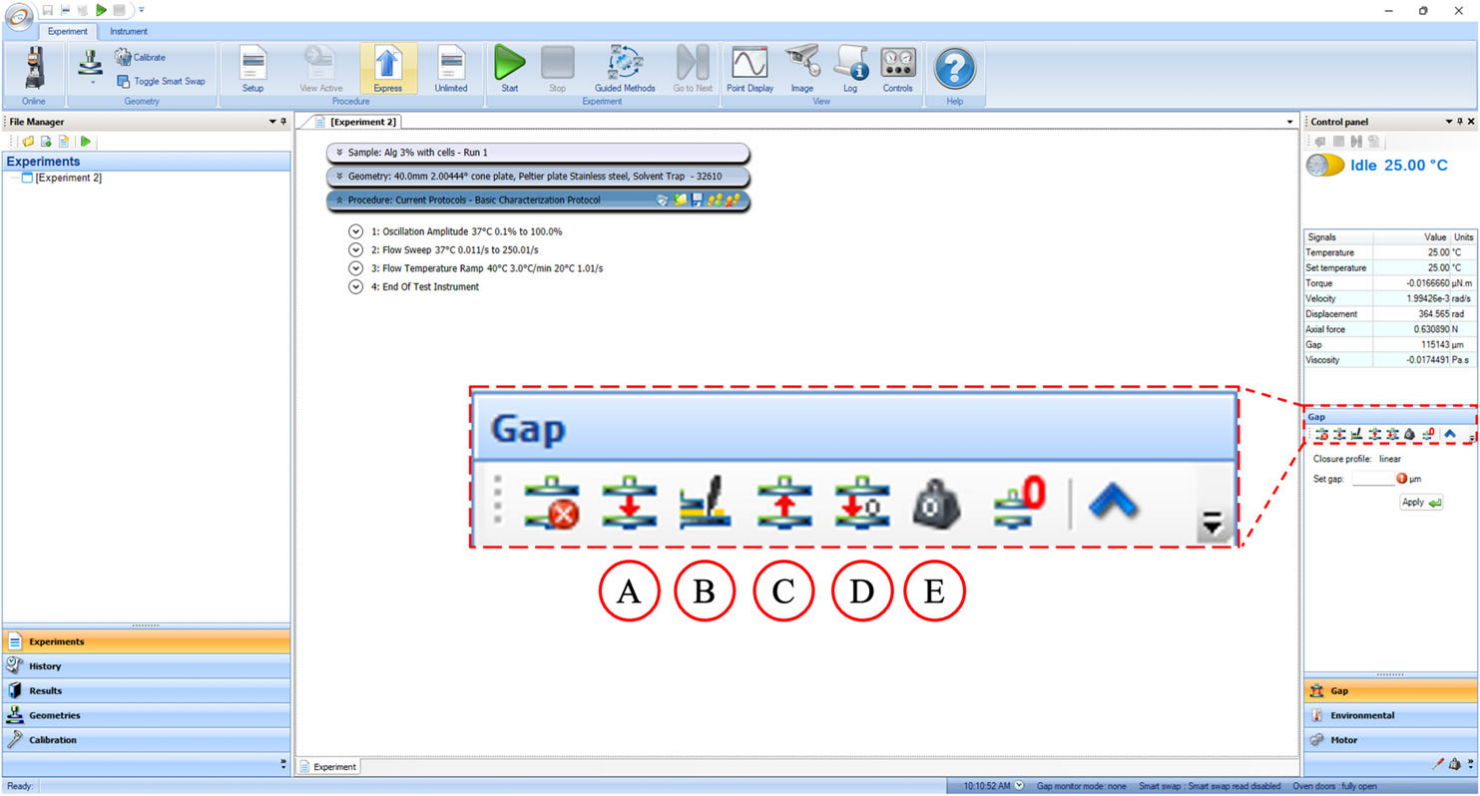
Software interface of TRIOS. (**A**) Go To Geometry Gap, (**B**) Go To Trim Gap, (**C**) Raise To Loading Gap, (**D**) Zero Gap, and (**E**) Zero Axial Force.

2 Turn on the main electronics box if it is not already on. Avoid turning this off as much as possible. Wait 30 min before proceeding.
3 Unscrew the black bearing clamp from the bearing. Turn on the heat exchange unit and computer.
4 Turn on the rheometer with the Power button (Fig. 3B). Wait until the main screen of the instrument turns on and open the TRIOS software on the computer to connect to the rheometer.
  The rheometer can be connected to when the circular symbol changes from gray to green.
5 Unless photosensitive materials are being used, turn on the LED light with the Toggle Light button to make it easier to see the testing area.
6 At the top, go to Experiment > Geometry > Toggle Smart Swap off.
  Turning on Smart Swap will allow the rheometer to automatically read the barcode on the geometry, but the cone does not have a solvent trap included, which is necessary to prevent water from evaporating in the samples. The solvent trap adds some parameters that need to be compensated for in the geometry calibration.
7 Screw the cone accessory (40‐mm diameter, 2° angle, 51‐µm truncated gap) on to the bearing. Under Experiment > Geometry, there is a dropdown arrow under the currently loaded geometry icon. Click the dropdown and select the geometry describing the cone with a solvent trap.
  The geometry will depend on the properties of the material. The cone uses less material and applies constant shear across the radius of the cone. The cone should not be used if the material has large particles, where the rule of thumb is that gap height should be 10× greater than the largest particle or accumulation. In this case, and in cases where the material is very low viscosity (it flows easily like water), a plate should be used.
8 The cone has a well on the top of the geometry. Fill this with water as it will be used later as an anti‐evaporation barrier together with a solvent trap.
  Depending on the reactivity of the material with water, an alternative is silicone oil.
9 Near the bottom‐right side of the screen, select the “Gap” tab and mouse over the symbols to view their function (Fig. 4).

10 Click on the symbol to Zero Axial Force. Then click on the symbol to Zero Gap.
11 Once the gap is zeroed, select Yes on the window that pops up to move the head of the rheometer back up.
12 Go to Experiment > Geometry > Calibrate. Only calibrate the categories if there is a yellow warning symbol. This can be done by clicking the dropdown button next to “Calibration” under the category and clicking the Calibrate button.
  The most common calibration will be “Rotational Mapping” that needs to be checked every 1 to 2 months.
13 Once the calibration is complete, click the “Accept” button. The rheometer is ready for testing materials.

#### Setting up the rheological experiments

14 There are three main experiments that should be conducted for any biomaterial used for printing: linear viscoelastic region (LVR) characterization; apparent viscosity characterization; and temperature‐dependent viscosity characterization.
  Most of the time, these three experiments can be run directly after the other. However, this mainly depends on the apparent viscosity characterization. The reason for this will be explained later, and the protocol to set up a series of three tests is described below. All tests will take ∼25 min to complete.
15 Near the bottom‐left of the screen, select the Experiments tab. Fill the blank fields of the Sample tab.
  Set the Name of the sample to describe the date, composition of the material being tested, and replicate. For example, “Jan1 ‐ 3% ALG + 0.5 mg/ml COL with cells ‐ Run 1”. This will make it easier to locate later under the “History” tab, found under the “Experiment”s tab on the bottom‐left side of the screen.
16 Click the dropdown button next to Sample > File Name and set the File Path to an easily locatable folder.
  Creating a sub‐folder in the easily locatable folder with the date or experiment will help organize the many data files to sort through later.
17 Expand the “Procedure:” tab in the same window to set up the series of experiments. Mouse over the symbols on the tab to view their function.
18 Make a new procedure by clicking the Remove All Steps symbol.
19 New steps can be created by clicking the Append Default Step symbol. Create three new default steps.
  By clicking on the name of the step next to the step number, the type of experiment can be changed.
20 Change the first step to an Oscillation Amplitude test. Set the Temperature to 25°C and Soak Time to 60.0 s. This is the test used to characterize the LVR, as well as the Storage and Loss moduli. Set the Frequency at 1.0 Hz and set the Strain % to test from 0.1% to 100.0% strain. Keep Points per decade at 5 unless more data is desired.
  There is always a slight delay in the response of the material to initial shear conditions and a lot of noise at low shear, so typically the data at under 1% strain is omitted during data analysis.
21 Change the second step to a Flow Sweep test. Set the Temperature at 25°C and Soak Time to 60.0 s. Set the Test Parameters to apply a Logarithmic sweep of Shear rate from 0.01 to 250.0 s^−1^.
  The shear rate can be set higher, depending on the material. Notably, if the shear rate is set too high for a given material, the sample may be expelled as the geometry rotates at higher rates. For most bioprinting‐related biomaterials, a shear rate up to ∼250 s^−1^ is sufficient to characterize the relevant rheological behavior. However, if the goal is to obtain a comprehensive understanding of material rheology, the shear rate can be set to ≥1000 s^−1^.
22 Change the third step to a Flow Temperature Ramp test. Set the Start temperature to 39°C, Soak time to 120.0 s, Ramp rate to 3.0°C/min, End temperature to 20°C, and Soak time after ramp to 0.0 s. Test Parameters should be set to a Shear Rate of 1.0 s^−1^ and Sampling interval to 1.0 s/pt.
  It is generally easier for the rheometer to decrease the temperature and record accurate measurements than the opposite. If desired, the Ramp rate can be decreased further for more precision but that will take more time.
23 Save this procedure in the folder. This procedure can be loaded for future materials.
24 Look at the main instrument and clean the bottom plate (an advanced Peltier plate) with a wet paper towel. Ensure that there is as little dust as possible on both the cone and the plate.
  If cells are in the sample, wipe the plate with 70% ethanol.
25 Load the sample on the bottom plate by simply pouring the sample directly out of the tube. A cone geometry requires only 0.6 ml of sample.
  Only use a syringe or spatula as a last resort to transfer material on to the plate as doing so adds unwanted stress in the material. These types of materials have “memory”, and the results obtained from tests will contain this stress, resulting in less‐than‐accurate measurements. However, if only a small amount of material was prepared, high surface tension will make it difficult to transfer. In this case, make sure to store the tubes lying on their sides let gravity distribute the material more evenly.
26 Fit the solvent trap housing on the plate.
27 Do not worry if too much material was poured out because the material must be trimmed anyway to decrease the effect of drag on the walls of the sample.
28 Lower the geometry by selecting the Go to Trim Gap symbol. Lock the bearing from rotating by pressing the Bearing Lock button on the front panel of the instrument.
  This will make it easier to trim the material and prevent the cone from adding shear stress while trimming.
29 Use a quarter of a weighing boat or a metal trim tool as a straight edge to remove any excess material; a plastic weight boat is preferred so that there is no risk of scratching the Peltier plate. This can be done by aligning the straight edge of the selected tool flush with the edge of the cone. Then flatly pull the tool toward the front of the instrument, gently scraping away material on each pass. Do this around the circumference of the cone.
30 Go back to the software panel and select Go to Geometry Gap. Some material will bulge out between the cone and plate, but do not trim this. This is the proper appearance of the sample for testing.
31 Fit one half of the solvent trap inside the housing, resting the lip of the trap in the filled well.
  If the water does not touch the lip of the trap, fill the well with a little more water, just until the lip touches the surface of the water. If the well is over‐filled use some paper towel to absorb the excess.
32 Fit the remaining half of the solvent trap into the housing. The system is ready to run tests.
  If a spatula was used to transfer the material, the Soak Time of the first step should be increased to at least 180 s to allow it to rest longer for more consistent results.
33 In the software, at the top of the screen, select the Experiment tab and start the experiment by selecting the green Start symbol. The Results screen will automatically open when the experiments begin running to display real‐time recorded data.
34 When the procedure is complete, the system will display “Idle” in the Control panel on the right side of the screen. Carefully remove the solvent trap from the housing.
35 Raise the geometry by selecting Raise to Loading Gap on the software.
36 Lock the geometry and use disposable paper towels to clean the sample off both the plate and cone. Use distilled water or 70% ethanol to clean the surfaces as much as possible.
37 Repeat the experiments at least two more times if possible.

## Basic Protocol 3 SCAFFOLD DESIGN AND BIOPRINTING

This protocol describes the complete workflow for preparing printing solutions and bioprinting tissue constructs. It includes the key steps for evaluating print quality and supporting cell‐recovery post‐printing. The protocol is organized into four main sections as follows:

Steps 1 to 15 outline the preparing of printing solutions, including 0.1% polyethyleneimine (PEI) and a crosslinker solution (0.1% PEI and 50 mM CaCl_2_).
Steps 16 to 30 provide step‐by‐step instructions to the reproducible bioprinting process, including scaffold design and calibration pre‐printing.
Steps 31 to 38 focus on fine‐tuning printing parameters through mathematical modeling and visualizing resulting printing quality using microscopy.
Steps 39 to 45 cover critical post‐printing procedures to help cells recover from bioprinting‐induced stress prior to downstream applications or experiments.

*NOTE*: Tapered dispensing needle tips are preferred for bioprinting because their design minimizes the shear stress experienced by encapsulated cells.

*NOTE*: PEI coating can have adverse effects on cell morphology, often causing them to remain round and inhibiting proper spreading. For constructs that retain their structure post‐printing, it is recommended to transfer the constructs to a new well plate without PEI coating for further culture to avoid these negative effects on cell behavior.

*CAUTION*: Printing parameter determination can be conducted with cell‐free solutions if using low density cell incorporation. For high density cell incorporation, it is recommended to determine parameters with a cell‐laden bioink solution.

*CAUTION*: When printing with cells do not exceed an extrusion pressure of 25 kPa to avoid cell damage.

*CAUTION*: Rinsing is important to remove residual crosslinking agents and unreacted components using suitable buffer/saline solutions. For example, sterile 1× PBS can be used if the construct materials are not sensitive to phosphate ions.

### Materials

H_2_O, distilled or Milli‐Q
Autoclaved polyethyleneimine (PEI) solution (0.1% w/v final; Thermo Scientific, cat. no. J61270.22)
Autoclaved calcium chloride (CaCl_2_) solution (50 mM final; Sigma‐Aldrich, cat. no. 223506)
70% ethanol
Autoclaved sodium chloride‐saline solution (NaCl) (0.9% w/v final; Sigma Aldrich, cat. no. S9888)
Cell culture medium

100‐ml Gibco bottle (Fischer Scientific, cat. no. 10339001)
Scale
Weigh boats
Spatula
Autoclave
0.2‐µm syringe filters, PES (Corning, cat. no. 09‐754‐29)
50‐ml Falcon tubes
12‐well culture plates (Fisher, cat. no. 08‐772‐29 or Thermo Scientific, cat. no. 12‐556‐005)
Parafilm
Cell culture incubator, 37°C, 5% CO_2_
BioScaffold Printer 3.2 (GeSIM, cat. no. BS3.2)
Tapered dispensing needle tips, 27G, ID 0.008‐in. (Jensen Global, cat. no. JG27‐1.25HPTTX)
GeSIM Robotics software V1.17.4.5053
Kimwipes (Fisher Scientific, cat. no. 06‐666A)

#### Day 1: Preparing printing solutions

##### Preparation of PEI solution for coating the culture plates

Polyethyleneimine (PEI) is applied as a coating on the culture plates for 3D‐printing alginate scaffolds to improve the adhesion of alginate filaments to the culture plate during printing (Mendoza García et al., 2017). This effect arises from the negatively charged surface of alginate and the positively charged nature of PEI, which generate strong ionic interactions that enhance scaffold stability and attachment.

1 Take a clean, autoclavable glass medium bottle with a cap and add the required volume of distilled or Milli‐Q water (e.g., 100 ml).
2 Weigh the required amount of viscous PEI (e.g., 0.1 g to prepare a 0.1% w/v solution) using a plastic weighing boat or container. Use a flat spatula to transfer the PEI from its container.
3 Transfer the weighed PEI into the bottle of water. Rinse any remaining PEI from the weighing boat using small amounts of water from the bottle to ensure full transfer.
4 Repeat the rinse as needed until no visible PEI remains in the weighing container. Mix the solution thoroughly with a spatula until the PEI is fully dissolved.
5 Autoclave the capped bottle to sterilize the solution.
6 Once cooled, filter the solution using a 0.2‐µm syringe filter into a 50‐ml Falcon tube.
  Filtration is optional but highly recommended to remove any fibers or particles that will interfere with downstream imaging.
7 Dispense the PEI solution into sterile culture plates, adding just enough to cover the entire bottom surface (e.g., 1 ml per well in a 12‐well plate or 2 ml per well in a 6‐well plate).
8 Seal the plate with Parafilm.
9 Incubate overnight at 37°C in a cell culture incubator.

###### Preparation of printing solution containing PEI and calcium chloride

10 Take a clean, autoclavable glass medium bottle with a cap and add the required volume of distilled or Milli‐Q water (e.g., 100 ml).
11 Prepare a 0.1% (w/v) PEI solution. Refer to “Preparation of PEI solution for coating the culture plates” above.
12 Weigh calcium chloride powder on a weighing boat to make a 50 mM solution (e.g., for 100 ml of solution, weigh 0.735 g of calcium chloride).
13 Add the calcium chloride powder to the 0.1% (w/v) PEI solution and stir manually with a spatula until fully dissolved.
14 Autoclave the solution and filter the cooled solution using a 0.2‐µm syringe filter into a 50‐ml Falcon tube. See the annotation in step 6.
15 Store it in the refrigerator until the day of printing.

#### Day 2: Scaffold design and 3D printing

16 Wipe the 3D printer device with 70% ethanol.
17 Turn on the UV light and let it run for at least 10 min.
18 Take the prepared bioink from Basic Protocol 1, step 73, and fix it into a cartridge heater using the designated holder and locking mechanism (refer to Video 1).
  Temperature of the cartridge should be set between 25° and 37°C (ideally 37°C) to ensure consistent printing performance and viability.

**Video 1:** Demonstration of BioScaffold Printer 3.2 printer setup and 3D‐printing, related to Basic Protocol 3 (steps 18‐30).

19 Warm the printing solution to 37°C.
20 Remove the coated plate from the incubator.
21 Discard the PEI from the wells.
22 Pipette enough printing solution into each well to fully cover the printed construct.
  It is recommended to discard the PEI from each well and fill each well with the printing solution one at a time, ensuring the solution reaches ∼37°C before adding to each well immediately before 3D printing.
  The amount of printing solution in each well depends on the height of the designed scaffold. A ratio of approximately 1:1 should be sufficient. For example, if you are printing a 5‐layer scaffold with 1‐mm height, use ∼1 ml of crosslinker solution.

##### Scaffold design

**Figure 5:**
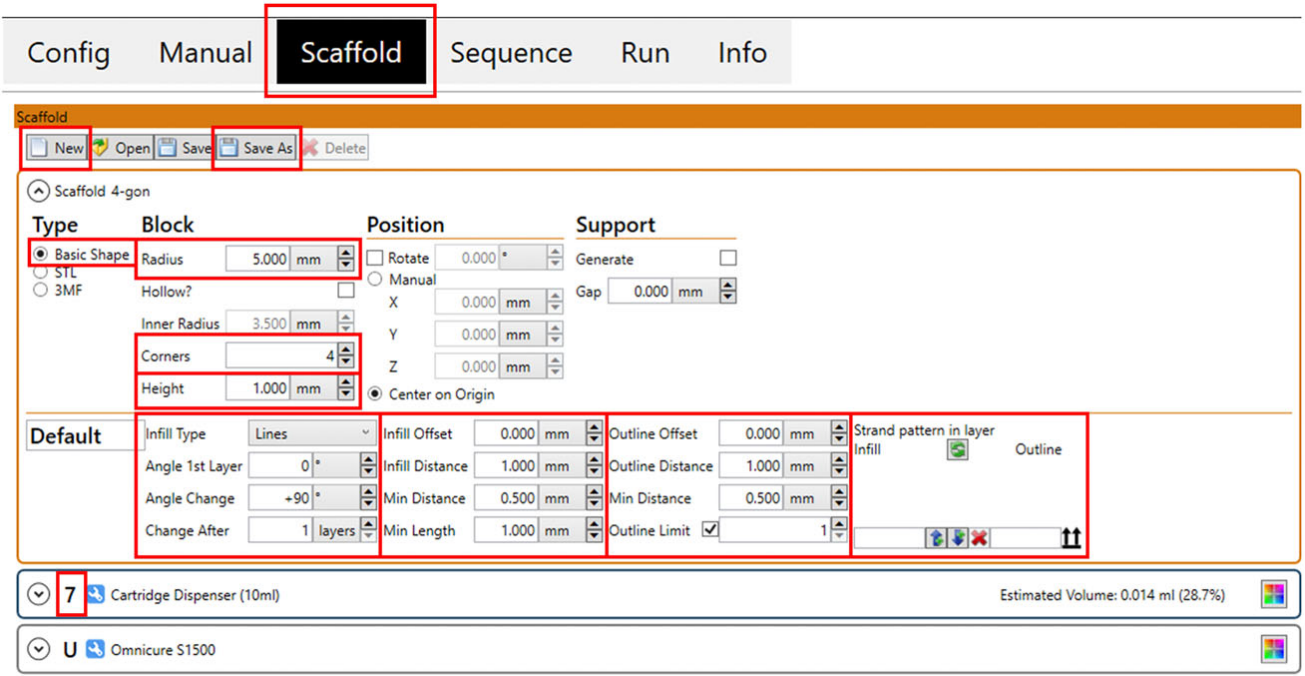
Required parameters for scaffold design (Basic Protocol 3, step 25).

23 Open the Gesim Robotics software.
24 Choose new file under the scaffold tab.
25 Select basic shape (Fig. 5).

**Figure 6:**
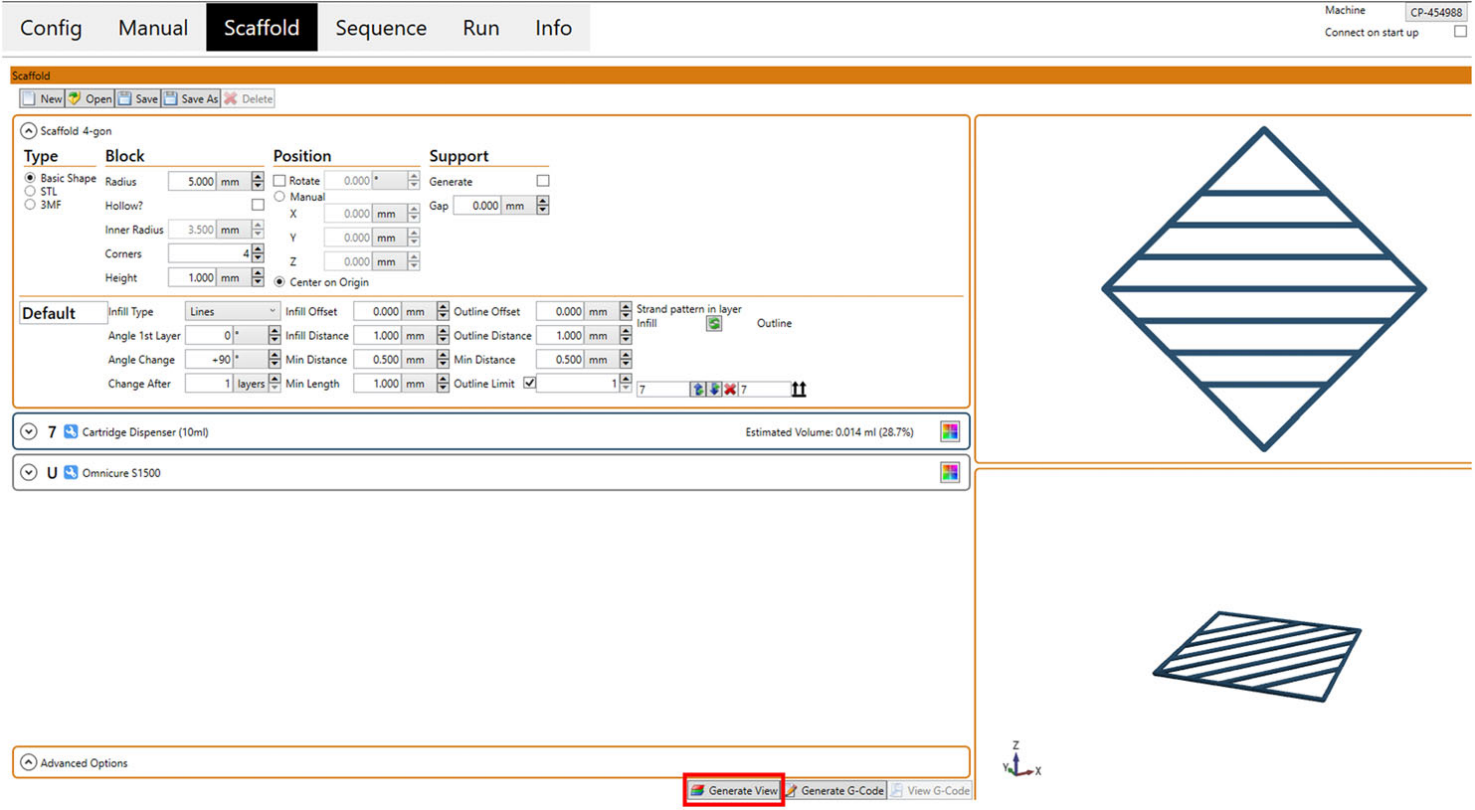
Generated view of example designed scaffold (Basic Protocol 3, step 28).

26 Enter desired parameters.
  Refer to Figure 5 for the basic required parameters.
  For a circular scaffold, increase the number of corners to obtain a rounded shape.
  Enter the desired cartridge dispenser number in strand pattern in layer (infill and outline). This indicates to the software which cartridge you would like to use to print the infill and the outline.
27 Click “Generate View” to see an image of the designed scaffold (Fig. 6).

28 Save the file.

###### Calibration and configuration

29 Refer to Video 1 for details on calibration and configuration.

#### 3D‐printing parameter determination

**Figure 7:**
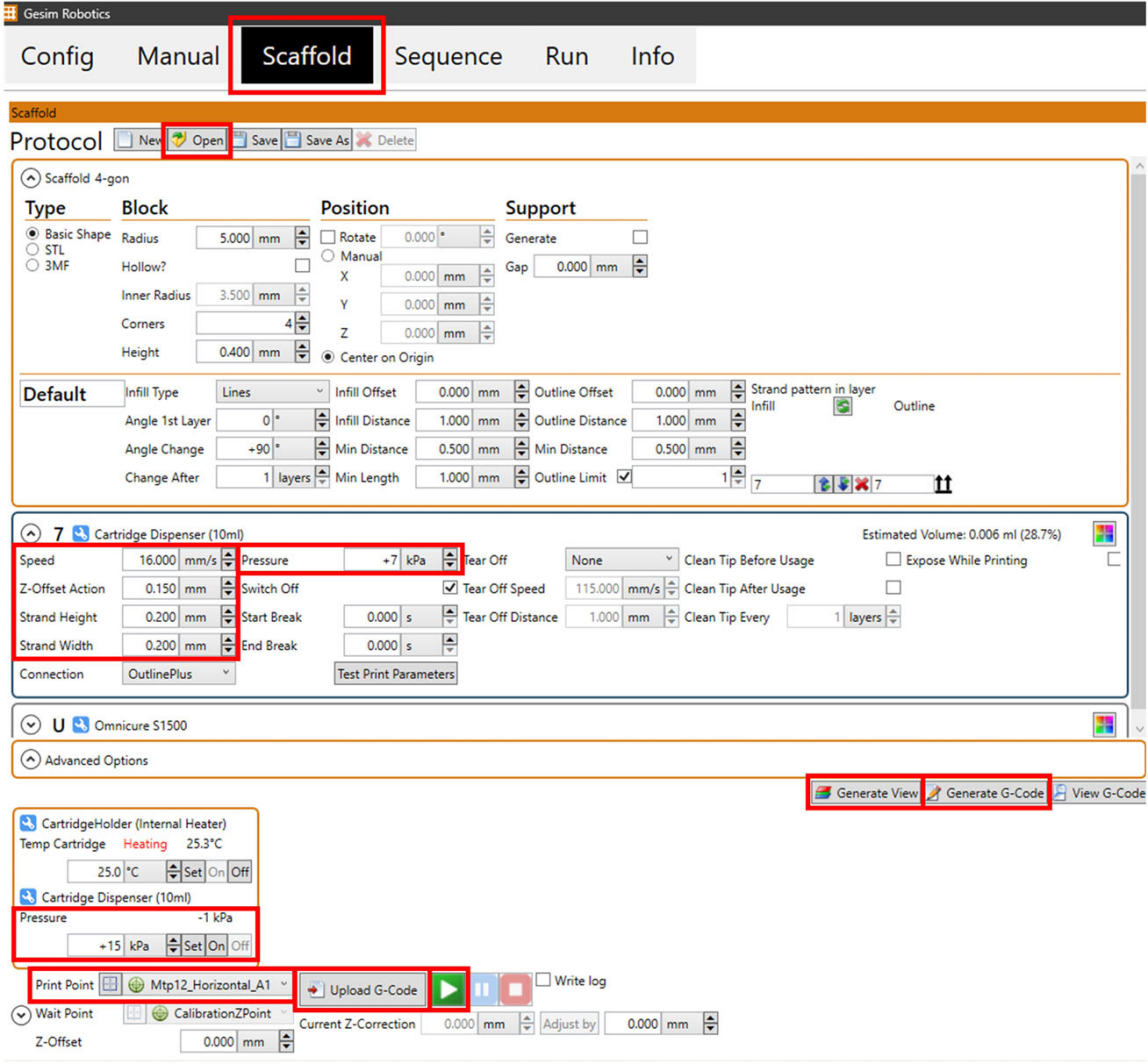
Example of printing parameter setup.

30 Open the saved scaffold design file on the Gesim Robotics software as highlighted in Figure 7.
  For initial printing, a simple 2‐layer scaffold design is typically sufficient.

31 Consult the literature to define an initial range of printing parameters based on your material. Choose a print point. Generate view > Generate G‐Code > Upload G‐Gode.
  Printing parameters are largely dependent on material viscosity and cell‐type. Examples of starting parameters from literature for alginate‐based materials are 8 to 25 kPa and 7 to 24 mm/s (Dahlan et al., 2025; Zimmerling, Aubrey, et al., 2024; Zimmerling, Zhou, et al., 2024).
  Generate view, generate G‐Code, and upload G‐Code must be done every time a parameter or print point is changed.
32 Set the extrusion pressure to slightly higher than your printing pressure and extrude a small drop of material to ensure the needle is not clogged. Wipe off excess materials using Kimwipes.
  An extrusion pressure of 5 to 10 kPa higher than printing pressure is usually sufficient. Refer to Caution above.
33 Press the play button to print the scaffold.
  Step 34 should be completed immediately after step 33. If prolonged time elapses (>1 min) repeat step 33 before completing step 34.
34 Image the scaffolds using the LionHeart Microscope Manual Mode. Use Brightfield imaging mode and select appropriate boundary conditions to image the full scaffold. Select montage.
  In cases where scaffolds have very obviously poor printability based on visual examination, microscopic imaging is not necessary. Examples of obviously poor printability include large amounts of material clumping with no discernable filaments, inadequate material extrusion with minimal printed filaments, and no filament attachment on the well.
35 Use the measure tool in the software to measure the scaffold diameter and pore size and adjust the printing parameters accordingly.
36 If necessary, continue in a trial‐and‐error manner to determine appropriate printing parameters based on desired printability.
  It is possible to have a range of appropriate printing parameters instead of a single set of parameters. In general, increased pressures will result in additional material extruded and will have a larger influence on printability as compared to speed.
37 Export final microscopic images with an appropriate scale bar for subsequent analysis in jpeg or png format.

#### Post‐printing procedure

38 After the scaffold is fully 3D printed, incubate it in the same printing solution used for printing at room temperature for an additional 15 min to allow for crosslinking.
39 After crosslinking, rinse the scaffolds with either sterile saline water (0.9% NaCl) or culture medium (for constructs) at least three times. See Caution above.
40 For bioprinted constructs, add fresh culture medium enough to submerge the constructs and incubate for at least 1 hr to equilibrate the constructs.
41 Gently remove the constructs using two spatulas from the bottom of the plate, as they may be attached. Lightly tap the scaffolds on each side, then carefully lift each side with the spatulas.
42 Transfer the constructs into a new 12‐well plate. Refer to Note above.
43 Add fresh, pre‐warmed medium and incubate in the cell culture incubator.
  Use complete medium supplemented with 20% FBS to improve viability post‐printing, especially for sensitive or primary cells (Dahlan et al., 2025). Once the cells start spreading or proliferating, switch back to the standard 10% FBS. For long‐term culture, consider adding a low concentration crosslinker agents (e.g., 20 mM CaCl_2_) in the culture medium to preserve filament fidelity and overall structure without adversely affecting cell viability (Zimmerling, Aubrey, et al., 2024).
44 Change medium every 2 to 3 days, or before the next planned experiments.

## 3D‐PRINTING PARAMETER DETERMINATION

Mathematical models of the printing process can assist in determining initial printing parameters, such as printing pressure and speed. We recommend using these models in parallel with the 3D‐printing process (Basic Protocol 3, steps 31 to 38) to fine‐tune these parameters and enhance reproducibility. Preliminary experiments can be conducted using the pressure and speed determined based on the models to ensure the width of the printed filaments closely matches the designed filament diameter. This step is critical because complex interactions between the printing solutions and the crosslinking mechanism, such as swelling and buoyancy, can alter the filament diameter, making it differ from the intended design.

*NOTE*: See Chen (2025a); Dahlan et al. (2025); Li et al. (2011); and Tabil et al. (2025).

### Materials

See Basic Protocols 2 and 3

1 If the flow behavior of the solution has been characterized through rheological testing (see Basic Protocol 2, Setting up the rheological experiments), a power‐law model can be established along with the fitted parameters. Further, the following equation, applicable to a tapered‐needle printing, can be used to evaluate the volumetric flow rate of the printed material, *Q*:
(2) $$Q=\frac{\pi D_{i}^{3}D_{o}^{3}}{32}{\left[\frac{3n\Delta P\left(D_{i}-D_{o}\right)}{4KL_{t}\left(D_{i}^{3n}-D_{o}^{3n}\right)}\right]}^{1/n}$$
  where *D_i_*, *D_o_*, and *L_t_* represents the needle dimensions of inlet diameter, outlet diameter, and length of tapered section, respectively; *ΔP* is the printing pressure as set by the operator, *K* the power law consistency index, and *n* the power law flow behavior index. The power law variables (i.e., *K* and *n*) can be obtained from the rheological data by fitting the stress‐strain relationship the power‐law model.
  Needle dimensions can be obtained from the manufacturer or by manual measurements. The needles used for this protocol had D_i_, D_o_, and L_t_ of 4.8 mm, 0.20 mm, and 31.9 mm, respectively.
2 The volumetric flow rates (*Q*), as evaluated above for the operator‐set printing pressure, can then be used to determine the stress‐free horizontal moving speed of the needle, or simply the printing speed (*v*) required to produce filaments with the designed diameter (*D_f_*), at the operator‐set pressure:

(3) $$v=\frac{4Q}{\pi D_{f}}$$

As such, the printing speed is directly dependent on the printing pressure. Repeating the above for each operator‐set pressure will result in a list of printing pressures and corresponding printing speeds.

## Basic Protocol 4 PRINTABILITY AND CELL VIABILITY ANALYSES, AND IMMUNOFLUORESCENCE ASSAY

This protocol outlines a step‐by‐step guide for assessing printability and seeding cells onto printed scaffolds. It also includes procedures for live/dead staining, imaging, and quantitative analysis using ImageJ software, as well as immunofluorescence assay for bioprinted constructs. The protocol is organized into five main sections as follows:

Steps 1 to 6 evaluate the printed constructs under microscopy to characterize printability.
Steps 7 to 24 describe the cell seeding technique to achieve uniform cell distribution and attachment. In bioprinting, some cell types are sensitive to printing parameters, such as shear stress and pressure, which can compromise their viability. However, these cells are often essential for creating the native tissue cellular arrangement. To address this, a cell seeding protocol is outlined for printed constructs, thus enables delicate or sensitive cells to be introduced post‐printing while preserving the overall structural integrity of the scaffold.
Steps 25 to 44 detail the preparation of stain solutions for the live/dead assay, including instructions for imaging the stained constructs using a fluorescent microscope.
Steps 45 to 61 cover the image visualization and quantitative analysis using ImageJ to determine the number of viable and non‐viable cells.
Steps 62 to 123 outline the immunostaining procedure for bioprinted constructs and cell‐seeded scaffolds.

### Materials

Gibco trypan blue solution, 0.4% (Fisher Scientific, cat. no. 15250061); store at room temperature
Complete culture medium specific to cell seeding type
Printed scaffolds in 12‐well plate (see Basic Protocol 3)
Collagen I, high concentration rat tail, 3 to 4 mg/ml (Fisher Scientific, cat. no. CB35249) neutralized with 1 M NaOH (see Current Protocols, 2006); store at 4°C
Live/Dead stain solution (see recipe)
Gibco 1× PBS, pH 7.4 (Fisher Scientific, cat. no. 10010023); store at room temperature
4% paraformaldehyde in PBS (Thermo Scientific Chemicals, cat. no. J61899‐AK); store at 4°C
Blocking/permeabilization solution for immunofluorescence (IF) (see recipe)
Primary antibody cocktail for IF (see recipe)
Hanks' balanced salt solution (HBSS), no calcium, no magnesium, no phenol red (Fisher Scientific, cat. no. 14175095)
Secondary antibody cocktail for IF (see recipe)
2‐methylbutane (Fisher Scientific, cat. no. AA19387AP)
15% and 30% (v/v) sucrose in 1× PBS (Thermo Scientific Chemicals, cat. no. J65148‐36)
Tissue‐Plus O.C.T. compound (Fisher Healthcare, cat. no. 23‐730‐571)
Porcine gelatin, type A, gel strength ∼300 g bloom (Sigma‐Aldrich, cat. no. G1890); store at room temperature
Autoclaved polyethyleneimine (PEI) solution (0.1% w/v final; Thermo Scientific, cat. no. J61270.22)
Silicone oil
Glycerol‐based mounting medium, i.e., ProLong Gold Antifade mountant (Fisher Scientific, cat. no. P36930)
Clear nail polish

Microscopic image files (see Basic Protocol 3)
ImageJ software (Fiji) V2.16.0
Hemocytometer or automated cell counter
Centrifuge
Biological safety cabinet (BSC)
20‐, 200‐, and 1000‐µl micropipettes and tips
Cell culture incubator, 37°C, 5% CO_2_
12‐well culture plates (Fisher, cat. no. 08‐772‐29 or Thermo Scientific, cat. no. 12‐556‐005)
Serological pipettes
Pipette controller
Aluminum foil
Leica DMI6000 B automated microscope
Leica software v3.7.3.23245
Ice bucket with ice
Styrofoam box with dry ice
Spatula, micro, tapered, 8‐in. (Fisher Scientific, cat. no. 21‐401‐10)
Glass slides
Cryostat microtome (Avantik Biogroup, cat. no. QS 11)
Q‐tip
Pencil
Hydrophobic barrier pen, i.e., Advanced PAP pen (Millipore Sigma, cat. no. Z672548)

#### Printability analysis

See Naghieh and Chen (2021); Ning, Sun, et al. (2018); Soltan et al. (2019); Zimmerling, Aubrey, et al. (2024); and Zimmerling, Zhou, et al. (2024).

**Figure 8:**
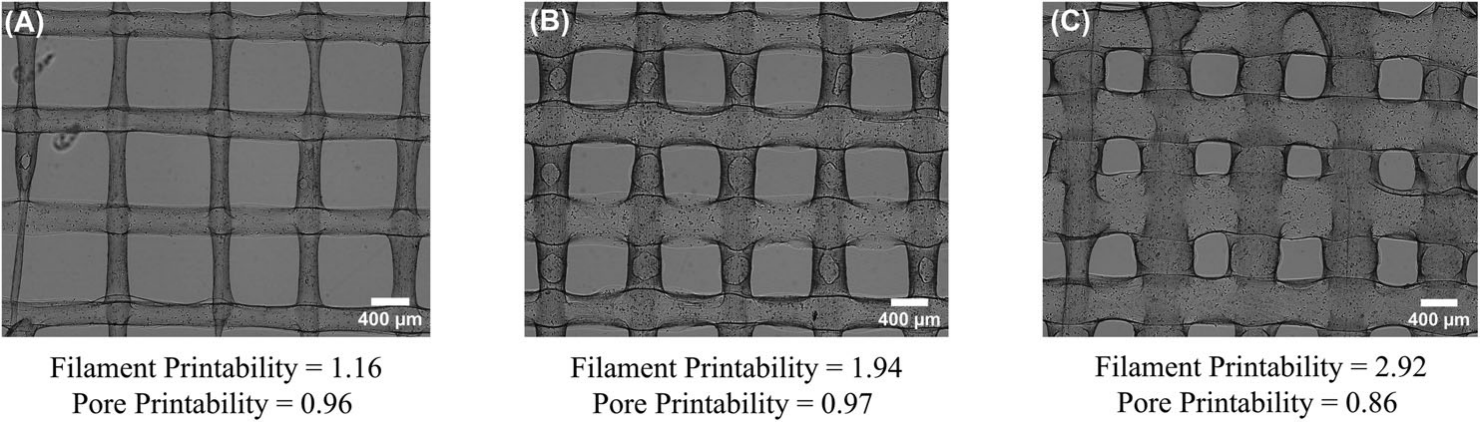
Example of (**A**) poor printability with thin strands, (**B**) desired printability, and (**C**) poor printability with thick strands.

1 Drag and drop a microscopic image file in png or jpeg format into ImageJ.
2 Use the line tool to draw a line across the scale bar. Set the scale by: Analyze > Set Scale > Enter known distance > Check global > OK.
  By checking global, it will set the scale for all images. If you do not check global, the scale will have to be set separately for each image.
3 Use the line tool to measure the filament diameter, press Ctrl + M to obtain the measurement. The diameter corresponds to the Length value shown in the Results window.
  Use a minimum of three measurements taken from three different areas of the scaffold.
4 Set results to provide area and perimeter by: Analyze > Set Measurements > Check area and perimeter > OK.
5 Use the polygon selection tool to select the pore size, press Ctrl + M to obtain the area and perimeter.
  Use a minimum of three measurements taken from three different areas of the scaffold.
6 Quantify printability based on filament diameter and pore size as shown in Figure 8 using Equations 4 and 5, respectively (Naghieh & Chen, 2021).

(4) $$Filament\;Printability=\;\frac{Experimental\;filament\;diameter}{Designed\;filament\;diameter}$$

(5) $$Pore\;Printability=\;\frac{{\left(perimeter\;of\;pore\right)}^{2}}{16\;x\;area\;of\;pore}$$

Equation 5 assumes square pore geometries.

#### Cell seeding

7 Complete Basic Protocol 1, steps 25 to 39 (Passaging cells).
8 Count the cells by mixing 10 µl of cell suspension with 10 µl trypan blue. Load 10 µl of the stained cells into a hemocytometer or automated cell counter to determine cell number and calculate the desired cell density.
9 Centrifuge 5 min at 300 × *g*, room temperature.
10 After centrifugation, carefully aspirate the supernatant without disturbing the cell pellet.
11 Resuspend the cells in fresh medium based on the cell count.
  Seeding density is dependent on cell type and will likely need to be determined from preliminary experiments. A density of ∼30,000 cells/cm^2^ has previously been used with bronchial airway epithelial cells (Zimmerling, Boire, et al., 2024) while ∼80,000 cells/cm^2^ has been used with HUVECs (Ketabat et al., 2023).
12 Retrieve the 12‐well plate containing the printed scaffolds from the incubator.
13 Place the 12‐well plate and neutralized collagen in the BSC.
14 Remove as much medium as possible from the well‐plate and discard.
15 Pipette 30 to 50 µl of neutralized collagen onto each printed scaffold.
  The exact amount of collagen is dependent on the size of the scaffold and may require preliminary experiments. Pipette gently onto the center of the scaffold, being mindful to avoid bubbles.
16 Incubate the 12‐well plate at 37°C and 5% CO_2_ for ∼10 min to allow the collagen to gel.
17 Place the 12‐well plate in the BSC.
18 Pipette 10 to 50 µl of cell suspension onto the center of each scaffold.
  The exact amount of cell suspension will depend on the size of the scaffold. Gently resuspend cells immediately before pipetting onto each scaffold.
19 Incubate at 37°C and 5% CO_2_ for 2 hr to allow the cells to attach.
20 Pre‐warm the medium. Refer to Caution in Basic Protocol 1.
21 Place the 12‐well plate and medium in the BSC.
22 Add enough medium to cover the entire scaffold in each well.
23 Maintain the plates at 37°C and 5% CO_2_.
24 Change the medium every 2 to 3 days. Refer to “Changing medium” in Basic Protocol 1.

#### Cell viability staining

Determine the volume of solution required based on how many wells you are studying and the height of the cell‐laden constructs or cell‐seeded scaffolds. Typically, 0.5 ml per well is sufficient to fully cover constructs/scaffolds <1 mm in height.

25 Prepare the Live/Dead stain solution in the BSC.
  Prepare the staining solution fresh on the day of the experiment, immediately before adding it to the constructs/scaffolds. Turn off the lights in the BSC while preparing the dye. The concentrations of the dyes in the medium may vary, but the key point is to maintain a consistent concentration throughout the study.
26 Pipette the stain solution to fully mix all components.
27 Ensure the lights of the BSC remain off during steps 25 to 38.
28 Retrieve the 12‐well culture plate containing the printed constructs from the incubator.
29 Place the 12‐well plate with the printed constructs, a fresh empty 12‐well plate, 10‐ml serological pipettes, and a pipette controller in the BSC.
30 Use the serological pipette to remove the medium from the 12‐well plate and discard the medium.
31 Transfer the constructs to the fresh 12‐well plate.
32 Wash the constructs with PBS once and discard the PBS.
  Ensure the PBS does not contain calcium/magnesium as this will degrade the samples. As an alternative, HBSS or culture medium may be used.
33 Pipette the stain solution into each well ensuring the whole construct is covered.
  After adding the dye to the constructs, wrap the plate with aluminum foil to avoid exposure to light.
34 Incubate at 37°C and 5% CO_2_ for 30 to 40 min.
  The incubation period of 30 min is approximate and is appropriate for cell‐seeded scaffolds. For cell‐laden constructs, the incubation time may need to be adjusted. You can image the stained samples while they are still in the staining solution. However, it is recommended to wash the constructs after checking the intensity to obtain higher‐quality images. If the intensity is insufficient, incubate for a longer period.
35 Retrieve the 12‐well plate from the incubator and place in the BSC.
36 Remove the stain solution from the 12‐well plate and discard the solution.
37 Wash once with PBS and discard the PBS.
  Ensure the PBS does not contain calcium/magnesium as this will degrade the samples. As an alternative, HBSS or culture medium may be used.
38 Add fresh PBS to each well to fully cover the cell‐laden constructs.
39 Turn the laboratory lights off.
40 Image the cell‐laden constructs using the Leica fluorescence microscope.
  It is recommended to use confocal imaging for cell‐laden constructs instead of a fluorescence microscope. This is because the constructs may have many layers of cells at high density on top of each other, which can be unclear when viewed under a fluorescence microscope.
41 Use brightfield and fluorescent channels with an exposure of 1 to 2 ms for the Brightfield channel and 200 to 400 ms for fluorescent channels.
42 Create a z‐stack of images by finding slightly above and below the top and bottom construct layers, respectively. Take images at three different locations of the scaffold.
  Imaging at three different locations is necessary if you do not have replicates. If you have at least three construct replicates imaging at one location that is representative of the entire construct is sufficient.
  The microscope does not have the ability to create a z‐stack for scaffolds >1 mm in height. In these cases, it is recommended to create a z‐stacks from the middle of the scaffold. Alternatively, multiple z‐stacks of 0.8 to 1 mm can be taken from multiple sections of the construct.
43 Export the images in the appropriate format for analysis (see Cell viability visualization and Cell viability quantification sections below).
44 Discard scaffolds after imaging.
  It is not recommended to image the same samples at multiple timepoints as this will affect the intensity of the dye. If you must image the samples later, it is recommended to store the samples at 4°C for no longer than 1 day after staining to prevent fluorescent degradation and keep them for no longer than 1 day after staining.

#### Cell viability visualization

45 Open the Leica software and load the desired project.
46 Choose a representative image within the z‐stack that clearly shows the live/dead cells.
47 Process the image by: Auto scale > Over/Under Expose > Drag the slider bar to adjust the exposure to minimize any blue hues > Drag the slider bar to adjust the background color > Grayscale > Adjust brightness level.
  Complete this step for red/green/brightfield channels separately.
48 Export the image with a scale bar in TIF format.
  Export red/green/brightfield channels separately.
49 Open ImageJ software.
50 Drag and drop the file into ImageJ software.
51 Create dashed lines on the image to visualize the printed strands: Select line tool > Draw a line on the border of the printed strand > Plugins > Dotted Line > Adjust thickness and dash type > OK.
52 Export the files in desired format.

#### Cell viability quantification

53 Export the images from the Leica software as an AVI file in overlay format.
54 Drag and drop the AVI file into ImageJ software. Click OK.
55 Choose a z‐stack range that has a clear view of the cells: Image > Stack > Z‐Project > Enter Range > Max Intensity > OK.
  This will merge the z‐stack into a single image with the max intensity of the pixels from each layer. If cells are obscured by subsequent layers perform the following steps on each z‐stack image individually.
56 Split the channels of the image: Image > Color > Split Channels.
  This will split the images into three windows of green, red, and blue as indicated in the brackets at the top of the window. Green and red will be used to determine the live and dead cells, respectively. The blue window can be closed.
57 If necessary, adjust the brightness and contrast of the images to provide a clearer image: Image > Adjust > Brightness/Contrast. Repeat step for both green and red channels.
58 Perform image segmentation to visualize the live and dead cells: Image > Adjust Threshold > Drag the slider bar to adjust the threshold > Apply. Repeat step for both green and red channels.
  Too low a threshold will merge cells together and too high a threshold will result in a loss of signal and missing cells.
59 Split any merged cells: Process > Binary > Watershed.
60 Count the cells: Analyze > Set the size range of the cells to remove any remaining noise > Check Summarize > OK. The count refers to the total number of cells.
61 Quantify the total number of cells based on the equation below.

(6) $$Cell\;Viability=\;\frac{\#\;of\;live\;cells}{\#\;of\;live\;cells+\#\;of\;dead\;cells}\times \;100$$

#### Immunofluorescence assay on whole cell‐laden constructs or cell‐seeded scaffolds

62 Remove culture medium from the constructs at the desired experimental time point.
63 Wash the scaffolds three times with an appropriate buffer. For alginate‐based constructs, options include HBSS, Dulbecco's PBS (without calcium/magnesium), or saline solution (see Current Protocols, 2006).
64 Fix the samples. In a fume hood, add 4% paraformaldehyde (PFA) to each scaffold.
  Ensure the volume is sufficient to fully cover each construct and maintain consistency across samples.
  From this point onward, procedures do not need to be performed in a BSC.
65 Incubate the constructs in 4% PFA for 1 hr at 4°C.
66 Prepare the blocking/permeabilization solution.
  Select your secondary host serum based on the secondary antibodies you are planning to use. For instance, if your secondary antibody is donkey anti‐rabbit, you should use donkey serum.
  Try to select all your secondaries from the same host but if you have two different species, you should use the sera of both the species (combine them).
67 Discard the PFA in the fume hood following appropriate disposal guidelines.
68 Add the blocking solution to each well.
69 Incubate the constructs with the blocking solution for 1 hr at room temperature.
70 Prepare primary antibodies in blocking solution (primary antibody cocktail).
  The required volume depends on the thickness of the scaffold and plate size. For example, in a 24‐well plate with a 3‐mm‐thick scaffold, 1 ml of antibody cocktail is generally sufficient if it fully covers the scaffold. Ensure consistency across all samples.
71 Add the prepared primary antibody cocktail to the constructs and seal the plate with Parafilm.
72 Incubate overnight at 4°C.
73 On the following day, prepare the secondary antibody cocktail using the same blocking solution used for the primary antibodies, which should have been stored at 4°C.
  To prepare 2 ml of secondary antibody cocktail for cTnT (anti‐mouse), we used donkey anti‐mouse Alexa Fluor 647 as the secondary antibody. This fluorophore allows visualization under the 647 nm wavelength (appears purple under the confocal microscope).
  Always include DAPI in the secondary cocktail to label cell nuclei. This is essential for distinguishing specific signals from background noise.
74 From this point forward, protect all samples and reagents from light. Keep them in a container covered with aluminum foil and cover the tube containing the secondary antibody cocktail with foil.
75 Wash the constructs three times with HBSS.
  For each wash, incubate the constructs in buffer for 5 min then discard the solution.
76 Add the prepared secondary antibody cocktail to the scaffolds.
77 Incubate at room temperature for 1 to 3 hr.
  This incubation time should be determined based on your experimental needs. You may also incubate the samples overnight at 4°C.
  For improved staining, place the plates on a tilting shaker with gentle agitation.
78 Discard the secondary antibody cocktail.
79 Wash the scaffolds three times with HBSS, 5 min each time.
80 Add fresh HBSS to the scaffolds and keep them on ice until ready for imaging.

#### Immunofluorescence assay on sliced cell‐seeded scaffolds

81 Repeats steps 62 to 65.
82 Wash the scaffolds three times with the same buffer used previously.
83 Incubate the scaffolds in 15% sucrose solution for 1 hr at 4°C.
84 Discard the 15% sucrose and incubate in 30% sucrose solution for 1 hr at 4°C.
85 In a small Styrofoam box, add dry ice and pour 2‐methylbutane over it to chill the liquid for a uniform freezing.
86 Label a plastic mold of appropriate size and gently pour clear frozen section compound (O.C.T. compound) to fill half the mold, avoiding bubbles.
87 Using a flat spatula, place the scaffold into the mold.
88 Add more frozen section compound to fully cover the scaffold.
89 Transfer the mold onto the chilled 2‐methylbutane/dry ice mixture using a flat spatula.
90 Allow the mold to freeze completely until the embedding medium turns solid white.
91 Cover each mold with aluminum foil and place it in a separate Styrofoam box with dry ice until all scaffolds have been frozen. Once freezing is complete, store the molds at −80°C until sectioning.

#### Cryosectioning: Slide coating

92 Prepare a coating solution containing 0.5% (w/v) gelatin dissolved in 0.1% PEI.
93 Individually dip each glass slide into the solution three times, ensuring each dip lasts at least 30 s.
94 Incubate the coated slides overnight at 37°C.
  If not used immediately, slides can be stored at 4°C for up to 2 weeks.

#### Tissue sectioning

95 Set the cryostat microtome temperature to −25°C at least 1 day before sectioning to allow sufficient time for temperature stabilization.
96 Mount the sample securely in the cryostat microtome.
97 Insert a new blade into the blade holder.
98 Apply a small amount of silicone oil to the edges of the blade using a Q‐tip to facilitate smoother sectioning.
99 Set the section thickness to the desired value (e.g., 20 µm).
100 Label each slide with a pencil prior to mounting sections.
101 After cutting each section, immediately mount it onto a pre‐coated slide.
102 Keep the mounted slides in the cryostat microtome during sectioning to maintain low temperature.
103 Once sectioning is complete, place the slides into a labeled slide holder box.
104 Enclose the box in a sealed plastic bag and store at −80°C until staining.

#### Antibodies staining

105 Remove the slides from the freezer and let them reach the room temperature.
106 Prepare a blocking/permeabilization solution.
107 Using a hydrophobic barrier pen, draw a circle around the mounted samples and make sure to close the circle. This prevents the solution to wash away from the sample and keep the solution on the slide and on the sample.
  Do not draw the line too close or too far from the sample, enough to fit 200 to 250 µl of the solution.
108 Carefully place the slides horizontally in humidity chambers filled with water
  Avoid the slides from being wet with the water, they should not be in contact with water.
109 Add 200 to 250 µl of blocking/permeabilization solution onto each slide.
110 Incubate them inside the chambers for 1 hr at room temperature.
111 Prepare the primary cocktail solution based on the recipe.
  Use the same recipe but consider 200 to 250 µl for each slide instead of 1 ml that we use for the whole scaffold.
112 Gently drain the blocking/permeabilization solution from the slides by touching the corner of each slide with a piece of tissue paper.
  Avoid touching the samples directly to prevent damage or contamination.
113 Add 200 to 250 µl of the primary antibody cocktail to each slide. Be sure to include proper controls for antibody specificity:
  a. Slides with only primary antibodies (no secondary).
  b. Slides with only secondary antibodies (no primary).
    These controls help ensure there is no non‐specific binding. The only signal visible on these control slides should be DAPI, which stains the nuclei. If other signals appear, troubleshooting may be required for your antibodies or sample preparation.
114 Place the slides horizontally back into the humidity chamber, and incubate them overnight at 4°C.
115 The following day, wash the slides three times using an appropriate buffer solution (e.g., HBSS for alginate‐based constructs). To wash the slides:
  a. Carefully drain the primary antibody solution by gently touching a piece of tissue paper to the corner of the slide within the marked circle, making sure not to contact the sample.
  b. Add 200 µl of buffer solution to each slide and incubate for 5 min.
  c. Drain the buffer in the same manner as above.
  d. Repeat this wash step two more times for a total of three washes.
116 Prepare the secondary antibody cocktail using the same formulation described in step 73.
  Use 200 to 250 µl per slide, instead of the 1 ml used for whole scaffold staining.
  Be sure to add the secondary antibody solution to the control slides **without** primary antibodies. **Do not** add the secondary antibody to the controls that contain only primary antibodies.
117 Blot the slides as described in step 112.
118 Add 200 to 250 µl of the secondary antibody solution to each slide.
  From this point onward, it is recommended to avoid exposure to light to protect fluorophores.
119 Incubate the slides in the humidity chamber for 1 to 2 hr at room temperature.
  Incubation time may vary depending on the sample type and should be adjusted as needed.
120 Wash the slides three times with HBSS, as in step 115.
121 Apply one or two drops of a glycerol‐based mounting medium to the sample, depending on its size. Gently place a coverslip over the sample.
122 Seal the edges of the coverslip using clear nail polish.
123 Store the slides horizontally at 4°C, protected from light, until imaging.
  Allow at least 24 hr before imaging, as most mounting media require curing time to achieve appropriate resolution.

## REAGENTS AND SOLUTIONS

### Blocking/permeabilization solution for IF

For 5 ml:
0.05 g Cytiva HyClone bovine serum albumin (BSA) (0.1% w/v) (Fisher Scientific, cat. no. SH3057.01)
100 µl of 0.02% serum from the secondary antibody host species, typically donkey (Millipore Sigma, cat. no. D9663) or goat (normal goat serum) (Abcam, cat. no. ab7481) (0.02% v/v final)
5 µl Triton X‐100 (0.1% v/v final) (Millipore Sigma, cat. no. X100)
4.895 ml buffer base (HBSS or DPBS; Current Protocols, 2006)
Store up to 3 months at 4°C
  Since IF spans at least two days, the blocking solution can be prepared in advance on day 1, with any excess stored at 4°C for use on day 2.

### HUVEC complete cell culture medium

For 500 ml:
435 ml cell culture medium (Gibco Advanced DMEM/F12, 1×, reduced serum medium; Fisher Scientific, cat no. 12634010); store at 4°C
50 ml fetal bovine serum (FBS), heat inactivated (10% v/v final) (Fischer Scientific, cat. no. A5256801)
5 ml anti‐anti (1% v/v final, Gibco antibiotic‐antimycotic, 100×; Fisher Scientific, cat no. 15240062); store at –20°C; aliquot the reagent into working volumes and avoid freeze/thaw cycles
10 ml HAT supplement (2% v/v final, Gibco HAT supplement, 50×; Fisher Scientific, cat no. 21060017); store at –20°C; aliquot the reagent into working volumes and avoid freeze/thaw cycles
Store up to 6 months at 4°C

### Live/Dead stain solution

Prepare in a 15‐ml Falcon tube:
2 µM final concentration Calcein AM (Invitrogen, cat no. 65‐0853‐39); store at –20°C in the dark
4 µM final concentration propidium iodide (Invitrogen, cat no. P1304MP); store at 4°C
1× PBS (Current Protocols, 2006)
  Avoid making stocks or excess solution. Calcein AM will be hydrolyzed by the PBS after prolonged exposure making the solution ineffective. Discard any excess solution after use.

### Primary antibody cocktail for IF

*Single staining solution*
For 2 ml:
4 µl cTnT (cardiac troponin T) antibody (Fisher Scientific, cat. no. MA5‐12960)
1.996 ml blocking solution (see recipe)
*Dual staining solution*
For 2 ml:
4 µl cTnT (cardiac troponin T) antibody (Fisher Scientific, cat. no. MA5‐12960)
4 µl CD31 antibody (Abcam, cat. no. ab76533)
1.992 ml blocking solution (see recipe)
Always prepare a fresh staining solution for every experiment
  Refer to the datasheet of each primary antibody to determine the recommended dilution ratio for either immunofluorescence (IF) or immunohistochemistry (IHC).
  It is advisable to empirically determine the dilution ratios for your specific experimental conditions.
  Prepare the primary antibody cocktail in the blocking solution. For example, for single staining of cardiac troponin T (cTnT, anti‐mouse), we tested dilution ratios of 1:250, 1:500, 1:750, and 1:1000. The 1:500 dilution gave the best results in our cell‐laden constructs seeded with cardiac cells.
  Do not use multiple primary antibodies from the same species. For example, you may combine mouse anti‐, rat anti‐, and rabbit anti‐ antibodies, but not two or more from mouse or any single species.

### Secondary antibody cocktail for IF

Secondary antibody (or antibodies) at the appropriate dilution ratio
DAPI (Fisher Scientific, cat. no. D1306) at a 1:100 dilution; store at –20°C
Blocking solution to complete the total desired volume (see recipe)
  We typically use a 1:200 dilution for secondary antibodies but always consult the manufacturer's datasheet and relevant literature for guidance.
  To add DAPI, first prepare a 1:100 stock dilution. Then, use this stock to achieve a final 1:100 dilution in the cocktail.

## COMMENTARY

### Critical Parameters

#### Cell passaging

It is important to maintain consistent cell passage numbers across all bioprinting experiments. Variation in cell passaging, especially primary cells can significantly affect cell growth rates, behavior, and response to post‐printing damage. Once printing and culture conditions are established, it is recommended to prepare sufficient stocks of cells at the same passage number to make sure all bioprinting experiments use cells with uniform passage history.

#### Cell density

Determine the cell density based on Live/Dead assay in Basic Protocol 4 and make sure the cells are consistently distributed throughout the printed constructs. Cell overcrowding can lead to nutrient depletion, hypoxia, and increased cell death after long‐term culture. As mentioned before, use cells of the same passage number to reduce variability at the determined seeding density.

#### Bioprinting live cells

Ideally, bioink temperature should be maintained within a physiological or material‐specific range (20° to 25°C for most cell compatible hydrogels, or 37°C for thermo‐responsive materials like gelatin) to preserve cell viability and material consistency during printing. It is not recommended to print for an extended period (3 to 4 hr) as cell suspended in the bioinks experience continuous shear and pressure stress within the extrusion system. Instead, keep printing sessions as short as possible or perform printing in batches.

### Troubleshooting

See Table 1 for a troubleshooting guide for the preparation and characterization of alginate‐based bioinks for 3D bioprinting of cell‐laden constructs.

**Table 1:** Summary of the Potential Problems that May Arise for Each Protocol, Possible Causes and Recommended Solutions to Address Them

Protocol	Problem	Possible cause	Solution
Basic Protocol 1: Bioink preparation	Bubbles in hydrogels after mixing with cells	Mixing process was too vigorous	Gently tap the syringe to dislodge bubbles and let it sit for ∼2 min to allow the bubbles to naturally rise to the top
			Purging the materials for <10 s may help eliminate larger bubbles
Basic Protocol 2: Bioink characterization using rheology	Rheological characterization results are not consistent and illogical trends of data are present	The sample is drying on the plate	Use a solvent trap
		Too much stress was applied when transferring samples by using a spatula or pipette	Add more soak time, e.g., 5 min. Or apply a low constant pre‐shear (0.1‐1 s^−1^) to all samples and soak to ensure all samples start at similar conditions
	Flow sweep tests indicate a sharp drop‐off in stress or viscosity data as shear rate increases	The shear rate is too high, and the sample is being expelled from the testing area	Decrease both the lower and upper bounds of the applied shear rate
Basic Protocol 3: Scaffold design and bioprinting	Poor material attachment	Higher z‐offset	Recalibrate the cartridge dispenser on the printer and measure each well
			Attempt to manually adjust the z‐offset lower until the needle lightly scratches the plate
		The well plate was not properly coated with PEI	Ensure well plates are coated for a minimum of 24 hr prior to printing at 37°C
	Material not extruding from the needle	Clogged needle due to material viscosity or cell clumping	Increase the extrusion pressure while gently cleaning the needle with a Kimwipe; if the material still does not extrude, replace the needle and recalibrate the cartridge dispenser
			To minimize bioink loss, place an autoclaved beaker or any container filled with autoclaved water beneath the needle, and submerge the needle in the water while gently purging the bioink using consistent pressure
	Over‐extrusion or material dropping from the nozzle tip	Material viscosity/temperature issues (for temperature‐sensitive hydrogels)	Use rheology to evaluate the material viscosity
			Adjust printing parameters, i.e., speed, extrusion pressure, nozzle temperature
			Consider using an external 0.2‐µm syringe filter instead of the cartridge barrel piston
	The printing starts to fail once printing the last layers	The z‐offset or z‐offset action (the z‐offset between layers) needs to be adjusted	Increase the z‐offset or z‐offset action (you need to adjust the z‐offset if you change the z‐offset action) if the fibers of the first layer are too narrow
			Decrease the z‐offset if the fibers of the first layer are too wide
			Ensure that the fiber width and height are equal; the recommended value for the z‐offset is twice the fiber height (or width)
	Yellowing of medium in bioprinted construct culture	Acidification of medium, hypoxic environment or contamination by bacteria or fungus	Check pH of the hydrogel materials prior to adding cells using pH test strips
			Design scaffolds with appropriate porosity to improve circulation and nutrient movement
			Ensure all surfaces are properly sterile using 70% ethanol or UV light
Basic Protocol 4: Printability and cell viability analyses, and immunofluorescence assay	Low cell viability post‐printing	Filament thickness, printing pressure	While preparing the bioink, handle the cells gently during mixing
			Try to minimize the printing time
			Avoid storing the bioink for >3 hr
			Lower printing pressure might induce reversible changes to cell damage
			Increase supplementation (e.g., 2× FBS) to boost cell recovery
			Screen cells with higher resistance to printing pressure to achieve the best results
			Avoid keeping the constructs in the same printing plate due to the cytotoxicity of PEI
			Wash the scaffolds thoroughly (e.g., with culture medium) for at least three times after crosslinking
	Poor image quality	Some biomaterials may exhibit background autofluorescence that can interfere with imaging	Use scaffold‐only as a control to identify which filter causes interference
			Discard the staining medium and replace it with fresh medium
			Use a confocal microscope instead of a standard fluorescence microscope for improved imaging resolution and depth
	Poor signal	Thicker and denser scaffolds may require longer incubation times with the dye to ensure adequate penetration and staining	Increase the incubation time
			Incubate at room temperature

### Understanding Results

#### Rheology

**Figure 9:**
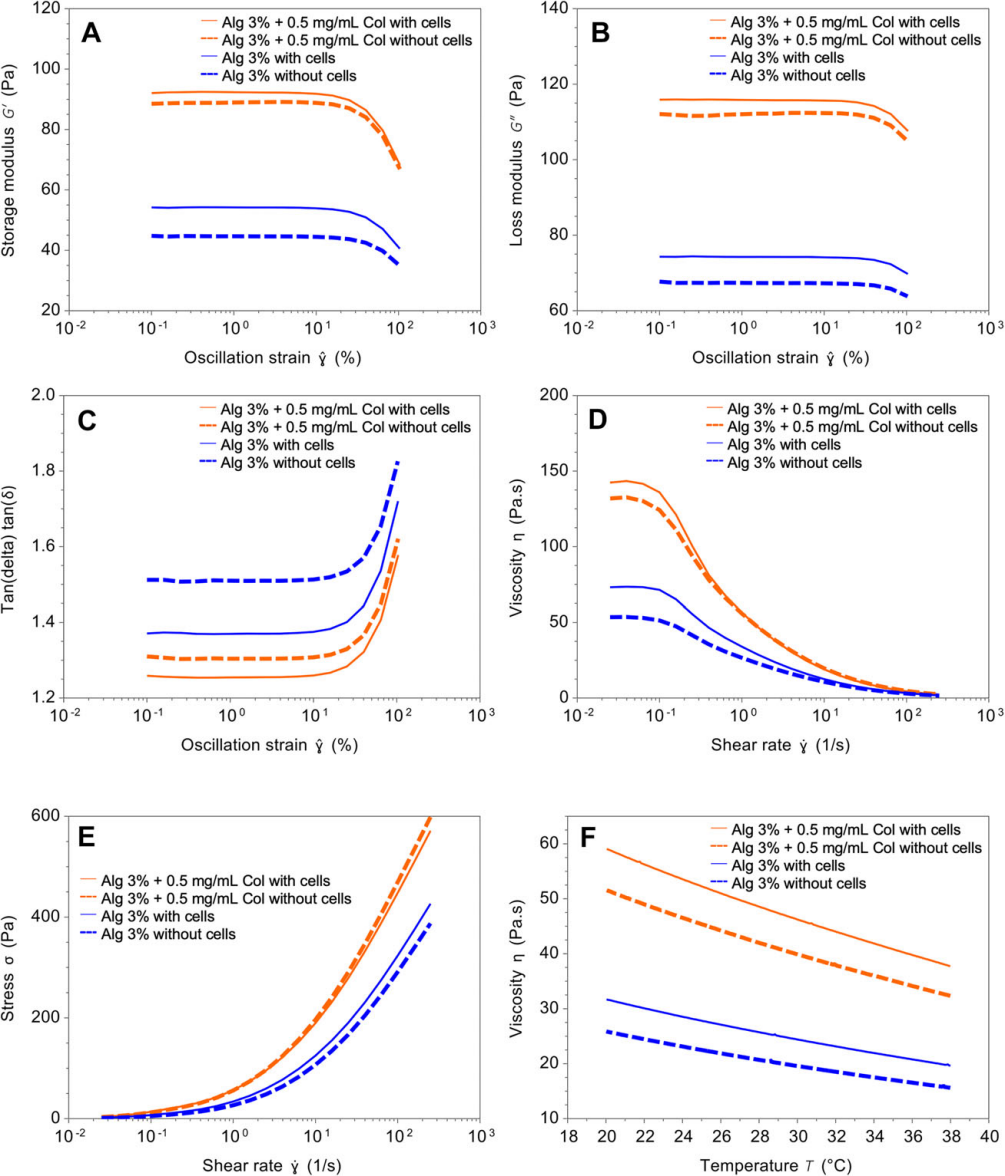
Examples of graphs and data typically received from rheological property characterization. Represented by orange and blue lines are the properties of previously printed biomaterial solutions, with and without cells. Panels (**A**‐**C**) show viscoelastic properties of the tested materials. Panels (**D**‐**E**) show the viscosity and shear stress response of the samples as the rate of shear increases. Panel (**F**) shows the viscosity profile of the samples as temperature changes at a constant, low shear rate.

The rheological properties of alginate‐based bioinks are influenced by factors, such as bioink concentration, cell density, and temperature. Using the rheological tests, as outlined in this protocol (via oscillation amplitude, flow sweep, and temperature ramp tests), one can iteratively tune and optimized bioink formulation for the high printing quality or fidelity and/or preserve cell survival. Figures 9A‐B show the storage and loss moduli, which indicate how “solid‐like” or how “liquid‐like” a bioink behaves, respectively. In oscillation amplitude tests, liquid‐like bioinks exhibit a dominant loss modulus, whereas solid‐like bioinks show a dominant storage modulus. These tests also give the information on loss angle (*δ*), with tan(*δ*) being the ratio of the loss modulus over the storage modulus. This ratio reflects the bioink ability to recover the deformation after force removal; tan(*δ*) <1 suggest that most deformation is recovered while tan(*δ*) >1 indicates that most deformation is permanent, manifesting as plastic deformation or flow. Bioink flow behavior or viscosity should be carefully adjusted to preserve the cell viability during the printing process, which can be achieved by modifying bioink composition or the cell density (Ning, Betancourt, et al., 2018). For example, Figure 9D show that adding both cells and collagen increase the viscosity of the alginate‐based bioink. Temperature also plays a critical role; as shown in Figure 9F, increasing temperature reduces viscosity. Overall, rheological tests simulate printing conditions and provide insights for refining bioink formulations to improve print quality and cell viability.

#### Printability

Printability of alginate‐based bioinks refers to its ability to form and maintain a 3D structure after printing and is essential for ensuring structural stability and supporting cellular functions. Printability of the bioink is dependent on rheological properties, which suggest the rheological behavior should be adjusted suitable for printing, as demonstrated in Figure 9. Scaffold design and printing parameters also influence printability and should be selected based on the intended application. Successfully fabricated scaffolds should demonstrate consistent filament deposition with interconnected pores and minimal breakages. Microscopic images as shown in Figure 8 combined with Equations 4 and 5 can be used to assess the filament diameter and pore sizes to determine printability. In addition to these quantitative measurements, factors including needle clogging, filament breakages, and printing consistency should be considered when determining printability. A filament printability value of 1 indicates the printed strands match the designed diameter; however, if this results in frequent breakages (as shown in Fig. 8A), a slightly larger value may be preferred for improved structural integrity. Similarly, a pore printability value of ∼1 is indicative of consistent pores with minimal irregularities and a stable structure. Notably, Equation 5 is only valid for square pore geometries, in cases where scaffolds have an angular design, printability may be assessed based on filament diameter and printing consistency.

#### Viability

**Figure 10:**
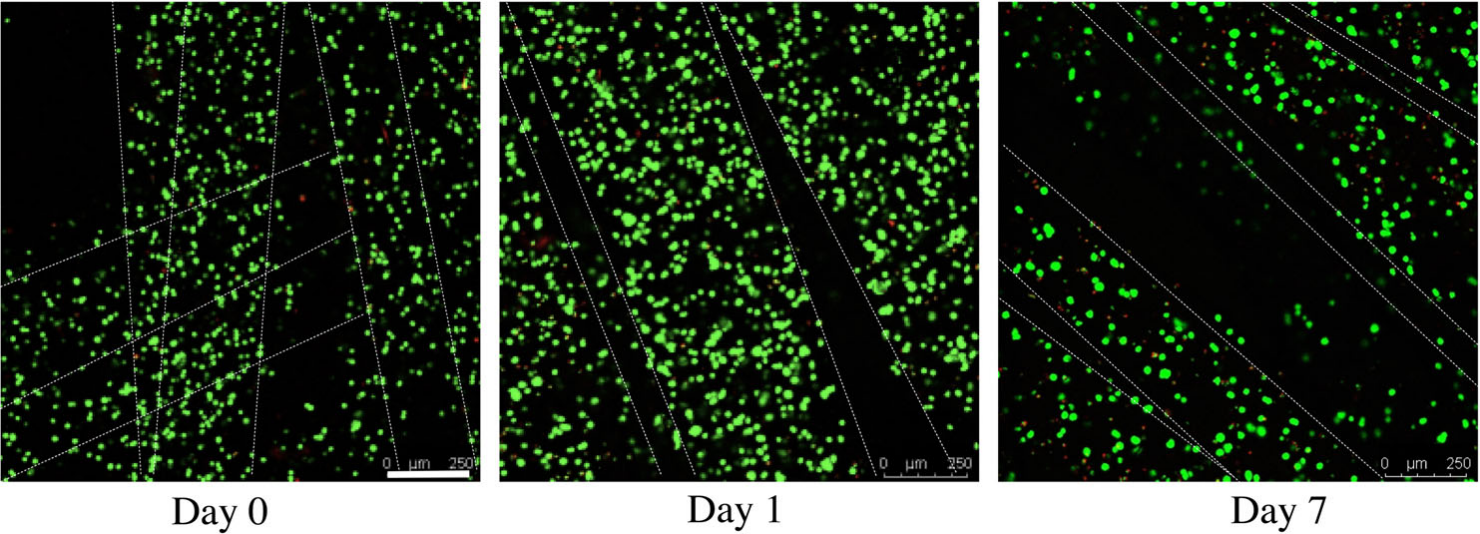
Example of 3D bioprinted HUVECs within a 16‐layer constructs on days 0, 1, and 7.

**Figure 11:**
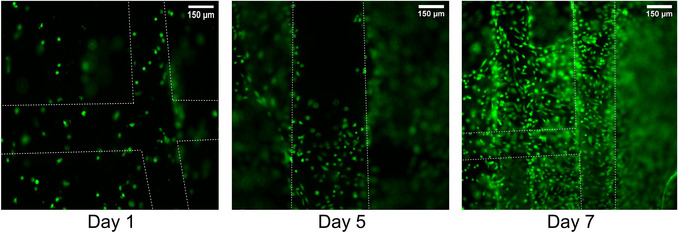
Example of seeded HUVECs on 2‐layer printed scaffolds at days 1, 5, and 7.

Cell viability provides insight into the biocompatibility and functionality of the printed/bioprinted models. Fluorescent microscopy provides a visual representation of cell viability with live cells indicated by green fluorescence and dead cells shown in red fluorescence (Figs. 10 and 11). Subsequent analysis of these images with Fiji software provides a quantification of cell viability. Fluorescent images should indicate evenly distributed cells for both seeded and bioprinted cells as indicated by Figures 10 and 11. A recovery period after seeded cells is to be expected with later timepoints demonstrating enhanced viability as demonstrated by Figure 11. Bioprinted cells may demonstrate a similar trend or have more consistent viability across timepoints depending on cell type as shown in Figure 10 (Xu et al., 2022). Appropriate image analysis in Fiji software (Schindelin et al., 2012) is necessary to obtain an accurate quantification of viability. Correctly interpreted images used for viability quantification will demonstrate cells with clear boundaries, minimal noise, and few cells omitted. A viability of ≥70% can be achieved under appropriate conditions for 3D bioprinted constructs (Ning et al., 2021; Xu et al., 2022), and a viability of ∼90% is expected with cell‐seeded scaffolds (Ketabat et al., 2023; Ning et al., 2016).

#### Immunofluorescence

**Figure 12:**
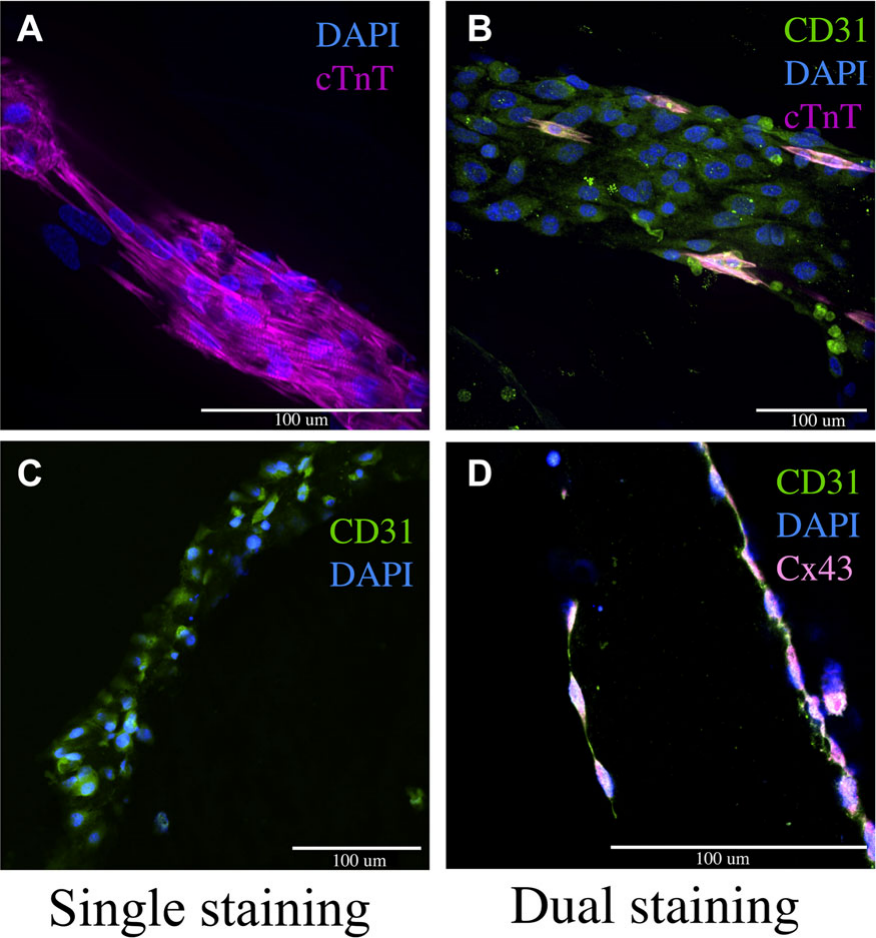
Representative immunofluorescence images of single and dual staining on alginate/gelatin scaffolds. Panels (**A**‐**B**) correspond to whole‐mount staining of constructs laden with HUVECs and seeded with cardiomyocytes. Cardiac troponin T (cTnT) staining identifies cardiomyocytes, while CD31, a cell–cell adhesion marker, labels HUVECs. Panels (**C**‐**D**) show sectioned constructs containing only HUVECs, stained for CD31 and connexin 43 (Cx43), a gap junction marker. Whole‐mount images (**A**‐**B**) demonstrate improved resolution and more integrated spatial localization of both cardiac and endothelial markers compared to the sectioned constructs (**C**‐**D**), which exhibit reduced spatial continuity. Nevertheless, both approaches revealed a vascularized organization of endothelial cells, achieving the primary objective of the staining.

Immunofluorescence is an important tool for detecting and localizing specific antigens within cells and tissue samples using fluorescently‐labeled antibodies, providing insights into the functionality of cells within constructs. Immunofluorescence can be performed using either the direct method (primary antibody only) or the indirect method (secondary antibody) (Im et al., 2019). In this protocol, we provided instructions for the indirect method. Staining can be performed either on the entire construct without embedding and freezing, or on embedded, frozen samples that are sectioned prior to staining (Fig. 12). The former allows for more integrated 3D localization of antigens within the construct, as it avoids potential damage caused by freezing and sectioning. This method offers higher spatial resolution in three dimensions but is limited by the fact that each whole construct can be used for only one staining, making it relatively expensive and unsuitable for repeated or multiplexed analysis. In contrast, frozen and sectioned samples may experience some signal loss or tissue damage, and the resulting images are limited to 2D slices. However, this approach enables multiple sections from the same sample, allowing for replication and the analysis of various antigens across different regions of the construct.

### Time Considerations

#### Basic Protocol 1: Bioink preparation

Cell thawing and expansion should be initiated at least 1 week prior to bioprinting to ensure cells reach a healthy state. Hydrogel mix can be prepared 1 to 2 days in advance and stored at 4°C, but prolonged storage is not recommended to maintain consistency and reproducibility.

#### Basic Protocol 2: Bioink characterization using rheology

Running rheology typically takes 1 to 3 hr, depending on the number of samples.

#### Basic Protocol 3: Scaffold design and characterization

The printing solution and 0.1% (w/v) PEI coating of culture plates should be prepared 1 day before bioprinting. Cell‐laden bioink should be prepared within 1 hr before printing to preserve cell viability and function. It is recommended to bioprint within 3 to 4 hr per session to minimize cellular stress from prolonged shear exposure.

#### Basic Protocol 4: Printability and cell viability analyses, and immunofluorescence assay

The cell viability assay typically takes up to 1 hr, depending on the number of samples. For immunofluorescence staining, allow up to 2 days as this includes incubation with both primary and secondary antibodies.

### Author Contributions

**Nuraina Dahlan**: Conceptualization; formal analysis; investigation; methodology; project administration; validation; writing—original draft. **Farinaz Ketabat**: Conceptualization; formal analysis; investigation; methodology; validation; writing—original draft. **Kathryn Avery**: Conceptualization; formal analysis; investigation; methodology; software; validation; writing—original draft. **Xavier Tabil**: Conceptualization; formal analysis; investigation; methodology; validation; writing—original draft. **Samira Khoz**: Conceptualization; methodology; writing—original draft. **Elise Altarriba**: Investigation. **Neeraj Dhar**: Funding acquisition; resources; supervision; validation; writing—review and editing. **Xiongbiao Chen**: Conceptualization; funding acquisition; resources; supervision; validation; writing—review and editing.

### Conflict of Interest

The authors declare no conflict of interest.

## Data Availability

The data that support the findings of this study are available from the corresponding author upon reasonable request.
